# Nature-Inspired 1-Phenylpyrrolo[2,1-*a*]isoquinoline Scaffold for Novel Antiproliferative Agents Circumventing P-Glycoprotein-Dependent Multidrug Resistance

**DOI:** 10.3390/ph17040539

**Published:** 2024-04-22

**Authors:** Alisa A. Nevskaya, Rosa Purgatorio, Tatiana N. Borisova, Alexey V. Varlamov, Lada V. Anikina, Arina Yu. Obydennik, Elena Yu. Nevskaya, Mauro Niso, Nicola A. Colabufo, Antonio Carrieri, Marco Catto, Modesto de Candia, Leonid G. Voskressensky, Cosimo D. Altomare

**Affiliations:** 1 Organic Chemistry Department, RUDN University, 6 Miklukho-Maklaya St, Moscow 117198, Russia; nevskaya.alisa@gmail.com (A.A.N.); avarlamov@sci.pfu.edu.ru (A.V.V.); arina.abydennik@gmail.com (A.Y.O.); lvoskressensky@sci.pfu.edu.ru (L.G.V.); 2 Department of Pharmacy-Pharmaceutical Sciences, University of Bari Aldo Moro, Via E. Orabona 4, 70125 Bari, Italy; rosa.purgatorio@uniba.it (R.P.); mauro.niso@uniba.it (M.N.); nicolaantonio.colabufo@uniba.it (N.A.C.); antonio.carrieri@uniba.it (A.C.); modesto.decandia@uniba.it (M.d.C.); 3 Institute of Physiologically Active Compounds of the FSBIS of the Federal Research Center for Problems of Chemical Physics and Medicinal Chemistry of the RAS, 1 SevernyiProezd, Chernogolovk 142432, Russia; anikina1970@gmail.com; 4 General and Inorganic Chemistry Department, RUDN University, 6 Miklukho-Maklaya St, Moscow 117198, Russia; nevskaya-eyu@rudn.ru

**Keywords:** pyrrolo[2,1-*a*]isoquinolines, Mannich bases, cytotoxicity, P-glycoprotein, inhibition, multidrug resistance reversal

## Abstract

Previous studies have shown that some lamellarin-resembling annelated azaheterocyclic carbaldehydes and related imino adducts, sharing the 1-phenyl-5,6-dihydropyrrolo[2,1-*a*]isoquinoline (1-Ph-DHPIQ) scaffold, are cytotoxic in some tumor cells and may reverse multidrug resistance (MDR) mediated by P-glycoprotein (P-gp). Herein, several novel substituted 1-Ph-DHPIQ derivatives were synthesized which carry carboxylate groups (COOH, COOEt), nitrile (CN) and Mannich bases (namely, morpholinomethyl derivatives) in the C2 position, as replacements of the already reported aldehyde group. They were evaluated for antiproliferative activity in four tumor cell lines (RD, HCT116, HeLa, A549) and for the ability of selectively inhibiting P-gp-mediated MDR. Lipophilicity descriptors and molecular docking calculations helped us in rationalizing the structure–activity relationships in the P-gp inhibition potency of the investigated 1-Ph-DHPIQs. As a main outcome, a morpholinomethyl Mannich base (**8c**) was disclosed which proved to be cytotoxic to all the tested tumor cell lines in the low micromolar range (IC_50_ < 20 μM) and to inhibit in vitro the efflux pumps P-gp and MRP1 responsible for MDR, with IC_50_s of 0.45 and 12.1 μM, respectively.

## 1. Introduction

**Figure 1:**
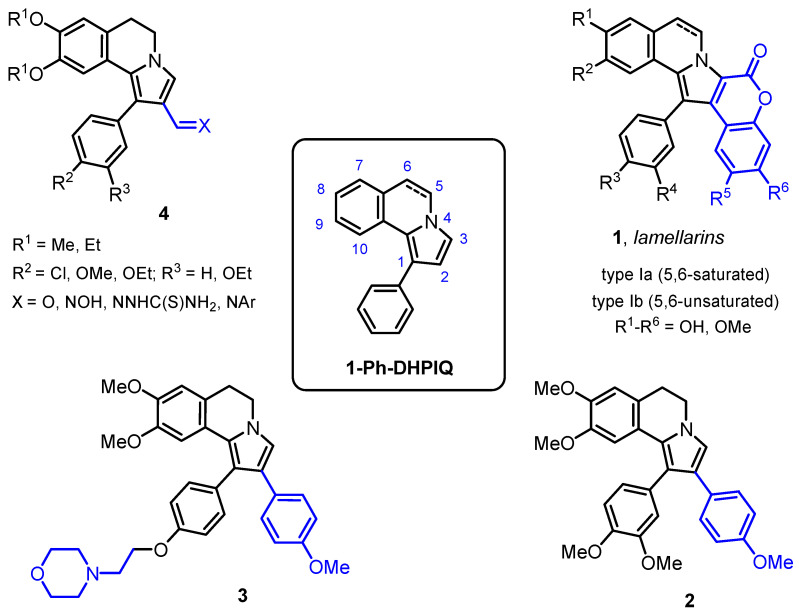
Structure of the 1-phenyl-5,6-dihydropyrrolo[2,1-*a*]isoquinoline (1-Ph-DHPIQ) scaffold and natural (**1**, lamellarins) and synthetic derivatives (**2**–**4**).

Pyrrolo[2,1-*a*]isoquinoline is an azaheterocyclic structure recurring in natural alkaloids isolated from marine invertebrates or plants, which are endowed with diverse biological activities [1]. Among them, type I lamellarins, incorporating into their structure R-substituted (R = OH, OMe) 1-phenyl-5,6-dihydropyrrolo[2,1-*a*]isoquinoline (1-Ph-DHPIQ, Figure 1), showed diverse biological activities, including anticancer and antiviral activities, as well as inhibition of the efflux pumps responsible for multidrug-resistance (MDR) [2]. In Figure 1, together with the general structure of lamellarins (**1**), the structures of synthetic bioactive 1-Ph-DHPIQ derivatives are shown, one of which (**2**) proved to be a potent topoisomerase I inhibitor [3], and the other (**3**), which merges the pharmacophore of tamoxifen (a well-known selective estrogen receptor modulator) with the 1-Ph-DHPIQ structure **2**, acting as an estrogen receptor (ER) modulator [4].

In previous studies, our research groups synthesized and tested for cytotoxicity against some diverse R-substituted 1-arylpyrrolo[2,1-*a*]quinoline derivatives (**4**, Figure 1) in tumor cells, along with 1-arylindolizines [5]. All the compounds held the aldehyde group in the C2 position and were tested as inhibitors of proliferation of rhabdomyosarcoma (RD), colon cancer (HCT116), adenocarcinoma of the cervix uterus (HeLa) and lung adenocarcinoma (A549). The choice of investigating the antiproliferative activity of our compounds on HCT116, HeLa and A549 cell lines was (and is) motivated by the prevalence of these types of cancer [6]. In contrast, rhabdomyosarcoma (RMS) is a rare but extremely aggressive form of sarcoma typically affecting children and young adults under the age of 20; the RD cell line appears suitable for testing in vitro new chemotherapeutic agents against RMS [7].

Some of previously synthesized 1-Ph-DHPIQ compounds, especially the hydrophobic aldehyde derivatives, like **4a** and **4c** (data in Table 1) and related imino adducts, proved to be cytotoxic in the tested tumor cell lines with IC_50_s in the low μM range [5,8]. Their mechanism of action has not yet been experimentally investigated, but in silico molecular docking calculations supported, likely as a part of a possible multitarget activity, the propensity of some suitably substituted 1-Ph-DHPIQ-2-carbaldehydes to bind the DNA-topoisomerase I complex [5], potentially blocking the DNA replication. The 5,6-dehydro analog of **4c** loses cytotoxicity (IC_50_s >> 100 μM), whereas several synthetic 1-Ph-DHPIQ-2-CHO derivatives were evaluated for their ability to inhibit P-glycoprotein (P-gp) and multidrug-resistance-associated protein-1 (MRP1) efflux pumps in MDCK-MDR1 (overexpressing P-gp protein) and MDCK-MRP1 (overexpressing MRP1 protein) cell lines and screened for their effects in drug combination assays with doxorubicin [9]. Compound **4c** and its closer analogs proved to be potent inhibitors of P-gp with IC_50_s < 0.5 μM and reversed MDR in tumor cells to doxorubicin even at low noncytotoxic concentrations. The lipophilicity of the substituents appeared to play a role in increasing the P-gp inhibitory potency of these compounds. In addition, some Schiff bases of 1-Ph-DHPIQs were disclosed as hits addressing Alzheimer’s disease-related target proteins, such as cholinesterases (ChEs) and monoamine oxidases (MAOs) [8].

In this study, maintaining the already explored R^1^, R^2^ and R^3^ substituents in structure **4** (Figure 1), we focused on the evaluation of possible replacements of the electrophilic aldehyde group in the C2 position, synthesizing and screening in vitro for their cytotoxicity in RD, HCT116, HeLa and A549 tumor cells, inhibition of MDR efflux pumps (P-gp, MRP1) and ADME-related physicochemical properties (e.g., water solubility, lipophilicity) of a number of 1-Ph-DHPIQ derivatives bearing C2 carboxylic groups (COOH, COOEt), nitrile (CN) and a morpholinomethyl moiety. The carboxyl group (carboxylate, ester) and nitrile [10,11] were examined as electrophilic groups less reactive than the aldehyde carbonyl [12], whereas the morpholinomethyl (Mannich base) derivative was synthesized in an early attempt at improving the aqueous solubility of the 1-Ph-DHPIQ derivative [13]. Within the limited molecular space examined, these chemical transformations allowed us to gain insights into the structure–activity relationships (SARs) of this class of nature-inspired molecules, especially in their inhibition of the MDR-related efflux pumps.

## 2. Results and Discussion

### 2.1. Synthesis

**Scheme 1:**
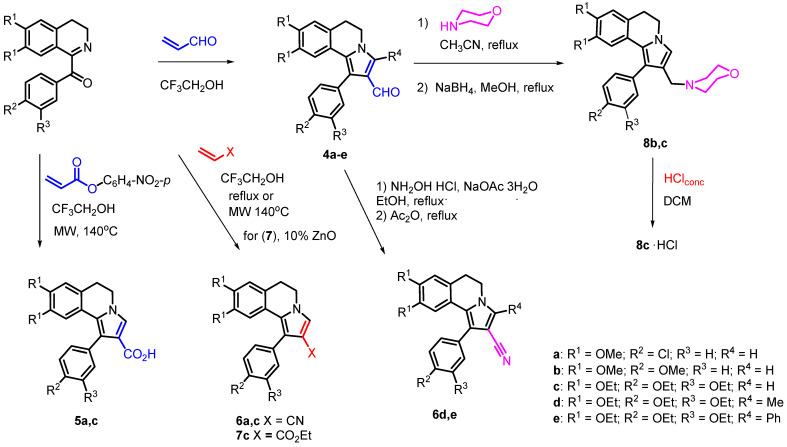
Synthesis of diversely R^1–4^-substituted 1-Ph-DHPIQ derivatives. Colors blue, red and pink highlight the new fragments/functional groups in C2 position.

The synthesis of new R-substituted 1-Ph-DHPIQ derivatives (**4b**, **5a**, **5c**, **6a**, **6c**, **7c**), bearing diverse functional groups at C2 (X = CHO, CN, COOH, COOEt), was carried out via a domino reaction between 1-benzoyl-3,4-dihydroisoquinolines and electron-deficient alkenes (Scheme 1). As reported earlier [14], the synthesis of the aldehyde **4b**, like the already reported **4a** and **4c**, was accomplished by reacting (3,4-dihydro-6,7-dimethoxyisoquinolin-1-yl)(4-methoxyphenyl)methanone with acrolein. Similarly, the carboxylic acids **5a** and **5c** were obtained by the reaction of the suitable 1-benzoyl-3,4-dihydroisoquinolines with *p*-nitrophenyl acrylate in 3,3,3-trifluoroethanol under microwave activation.

The carbonitriles **6a** and **6c** without the substituent R^4^ had been already synthesized from the suitable 1-benzoyl-3,4-dihydroisoquinolines in boiling trifluoroethanol [15]. The carboxymethyl ester **7c** was prepared by the reaction of the isoquinoline in the presence of ZnO (10 mol%) in trifluoroethanol under microwave activation. Suitable 1-Ph-DHPIQ-2-carbaldehydes **4** were used as starting materials for synthesizing either further carbonitriles **6d** and **6e**, or the Mannich bases **8b** and **8c** (Scheme 1). The one-pot reaction of aldehydes **4d** and **4e** with hydroxylamine hydrochloride in the presence of sodium acetate in EtOH, followed by boiling in acetic anhydride, led to the formation of carbonitriles **6d** and **6e**, respectively. The 2-morpholinomethyl Mannich bases **8b** and **8c** were synthesized by one pot reaction of aldehydes **4b** and **4c**, respectively, with morpholine in acetonitrile followed by reduction of the iminium intermediate with NaBH_4_ in MeOH. To increase the aqueous solubility of **8c**, it was converted to the corresponding hydrochloride salt.

### 2.2. Biological Evaluation

#### 2.2.1. In Vitro Cytotoxicity Screening

The newly synthesized 1-Ph-DHPIQs, along with the previously reported derivatives **4a**, **4c**, **6a** and **6c**, with camptothecin and doxorubicin as positive controls, were tested at the maximum concentration of 100 μM for their antiproliferative activity, using an MTT assay on RD, HCT116, HeLa and A549 cancer cell lines. Compounds showing more than 50% inhibition were tested at lower scalar concentrations, and IC_50_ values were calculated by interpolation of the dose-response curves (Table 1).

As shown by the antiproliferative activity data, among the newly synthesized compounds, only **8b** and **8c**, both carrying the 2-morpholinomethyl group, achieved noteworthy antiproliferative activity with IC_50_s << 100 μM. Of the two Mannich bases, the more lipophilic **8c** achieved 50% antiproliferative activity in all four cancer lines in the low micromolar range (IC_50_s < 21 μM). The newly synthesized DHPIQ-2-aldehyde derivative **4b** proved to be less active than the two more lipophilic congeners **4a** and **4c**. It appears quite clear that the replacement of the 2-CHO group with less electrophilic group, such as CN (**6a–e**), COOH (**5a**,**c**) and COOEt (**7c**), does result in a loss of activity.

#### 2.2.2. P-gp and MRP1 Inhibitory Potency

The P-gp inhibitory potency of eight DHPIQs was evaluated by measuring the transport inhibition of calcein-AM, a profluorescent P-gp substrate, in the MDCK-MDR1 cell line overexpressing P-gp. Moreover, to evaluate the MRP1 inhibitor activity, the same compounds were screened in MDCK-MRP1 cells overexpressing MRP1. MC18 [9] and verapamil [16] were used as positive controls for P-gp and MRP1 efflux pumps, respectively. The IC_50_ values (Table 2) show that five out of eight compounds tested (i.e., **5c**, **6c**, **7c**, **8b**, **8c**), regardless of the functional group at the C2 position, inhibited the P-gp efflux pump with a potency in the submicromolar range of concentrations (IC_50_ < 0.5 μM).

Regarding the substituents R^2^ and R^3^ onto the 1-phenyl group, it can be inferred that compared to Cl, OMe in the *para* position does improve the inhibition activity toward both the efflux pumps, whereas the EtO substituents in *meta* and *para* enhance potency toward P-gp (but not MRP1). Indeed, as far as P-gp/MRP1 selectivity is concerned, except for the tetraethoxy-substituted 1-Ph-DHPIQ carboxylic acid (**5c**) and nitrile (**6c**), which achieved a selectivity ratio toward P-gp of more than two orders of magnitude, the other compounds proved to be unselective (**4a**) or moderately P-gp-selective, with the tetraethoxy 1-Ph-DHPIQ Mannich base (**8c**) approximately 30-fold more active towards P-gp.

#### 2.2.3. Binding Affinity to Human Serum Albumin (HSA)

The interactions between the DHPIQ derivatives and HSA were evaluated by surface plasmon resonance (SPR), using warfarin, a well-known strong HSA binder, as a reference [17,18]. The assessment of the binding affinity to HSA can help to estimate the bioavailability in the early stage of drug design and development. By plotting the response at equilibrium, the dissociation constant (*K*_D_) value was determined for each test compound.

**Figure 2:**
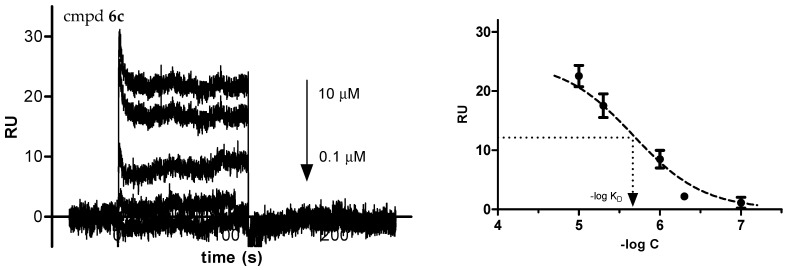

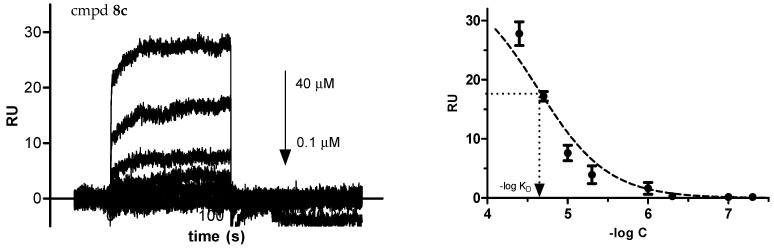
Surface plasmon resonance (SPR) sensorgrams (**left panels**), obtained at different concentrations of 1-Ph-DHPIQ derivatives **6c** (**top**) and **8c** (**bottom**) to immobilized human serum albumin (HSA); sigmoidal binding isotherms (**right panels**), with compound concentrations in logarithm scale. Response units (RU), expressed as the averages with SD from three repeated experiments, were fitted using nonlinear regression in GraphPad Prism software (vers. 5.01), and the log of the equilibrium dissociation constants (KD) was calculated by the curve interpolation.

The sensorgrams at different concentrations of all the DHPIQ compounds in Table 2 to immobilized HSA were recorded and the binding curves fitted to determine *K*_D_s. In Figure 2, the sensorgrams of the nitrile **6c** and the morpholinomethyl derivative **8c**, along with the corresponding binding isotherms, are shown. The binding curves of all the tested compounds showed that the responses at equilibrium were quite rapidly reached at any increasing concentration; therefore, the association and dissociation rate of interaction were too fast to be reliably determined.

Considering the average physiological HSA concentration in plasma (680 μM), at 10 μM concentration, compounds **4**–**7**, regardless the lipophilicity of the R-substituents, can be predicted to be highly bound to HSA, whilst the morpholinomethyl Mannich bases (**8b,c**) can be estimated to be HSA-bound for less than 40%.

### 2.3. Solvation-Related Parameters

The solubility of the investigated compounds was evaluated in PBS (50 mM, pH 7.4, 0.15 M KCl) at 25 °C using RP-HPLC as the analytical method (Table 2). The ester **7c** and the Mannich base **8b** were preliminarily monitored by RP-HPLC for the hydrolytic stability at pH 7.4 (50 mM PBS, 0.15 M KCl) and at pH 2 (0.01 M HCl_aq_, 0.15 M KCl), and found to be stable over 4 h at 25 °C in both conditions. All of the compounds proved to be poorly soluble in buffered water solution at neutral pH, even ‘practically insoluble’ (<0.1 mg/mL) according to the solubility categorization adopted by US and European Pharmacopoeia [19]. Nevertheless, the transformation of the aldehydes **4** into ethyl carboxylate ester **7c** or morpholinomethyl basic derivatives **8b**,**c** significantly improves the water solubility of the 1-Ph-DHPIQ analogs. In particular, the transformation of the DHPIQ-2-aldehyde via Mannich reaction (**8b**,**c**) opens the road to future developments of new water-soluble antiproliferative DHPIQ derivatives endowed with the ability to inhibit P-gp and reverse MDR.

To quantitate the effect of hydrophobicity on the biological activity of the examined compounds, a relative lipophilicity scale was determined by RP-HPLC [20]. The polycratic capacity factor (log *k′*_w_) for each compound in Table 2 was experimentally determined (details in the experimental section) and compared with 1-octanol–water partition coefficient calculated (Clog P) with ACDLabs software (release 10.0; Advanced Chemistry Development, Inc., Toronto, ON, Canada). Log *k′*_w_ values are quite well correlated with Clog Ps for the whole series (*r*^2^ = 0.791). However, omitting from the regression the two Mannich bases **8b** and **8c,** which, due to their basicity (p*K*_a_s about 6.9, estimated by ACDLabs software), should be predominantly in the protonated forms at pH 4.7 (i.e., the pH of the aqueous component of the mobile phase in RP-HPLC), the correlation slightly improves (*r*^2^ = 0.859), providing a linear equation with a slope equal to +0.8 and an intercept of about −0.1.

No linear (or even nonlinear) relationship was detected between the measured biological properties and the solubility/lipophilicity descriptors determined or calculated (Table 2) in this study, as shown by the squared correlation matrix of the determination coefficients (Table S1 in the Supplementary Materials). Lipophilicity should play a secondary role in modulating the anticancer cytotoxicity of the 1-Ph-DHPIQ derivatives (Table 1). The replacement of the electrophilic and electron-withdrawing CHO group in **4a** and **4c** with a less reactive electrophilic but more electron-withdrawing CN in the corresponding **6a** and **6c** derivatives resulted in a drop of cytotoxic activity, regardless of the hydrophobicity of the substituents onto the 1-Ph-DHPIQ scaffold. A drop of activity was also observed by replacing 2-CHO with COOH (**5c**) and COOEt (**7c**). Instead, with derivatives of the aldehyde group, such as the Mannich bases **8**, an increase in antiproliferative activity was achieved on all tumor cells tested. Between the two Mannich bases, the two- to four-fold greater activity of **8c** compared to **8b** apparently reflects the difference in lipophilicity of approximately 1.8 log P units. Similarly, the three- to four-fold greater cytotoxicity of **4a** compared to **4b** may be related to the difference of about one log P unit between the two aldehydes.

Even the inhibition data of the MDR-related efflux pumps P-gp and MRP1 (Table 2) did not significantly correlate with the lipophilicity parameter alone (either log *k′*_w_ or Clog P). First, the inhibitory potency on P-gp/MRP1 appeared to be not affected at all by the properties (electrophilicity, lipophilicity, bulkiness/polarizability) of the X functional group (CHO, CN, COOH, COOEt, MM). Sometimes the effect of lipophilicity is apparent from pairwise comparisons of substituents onto the 1-Ph-DHPIQ scaffold, and such an effect is manifested differently on P-gp (direct correlation) and MRP1 (inverse correlation). For instance, all the compounds bearing as R^1^ (8 and 9 positions), R^2^ (4′ position) and R^3^ (3′ position) the more lipophilic OEt groups, instead of OMe or Cl (as R^1^ and R^2^), achieved the higher inhibition potency against P-gp (IC_50_s < 0.5 μM). Besides these ‘local’ hydrophobic effects of the substituents, it is worth mentioning that the most water-soluble molecules **7c** and **8b**,**c** were among the most potent P-gp inhibitors.

### 2.4. Molecular Docking Calculation

**Figure 3:**
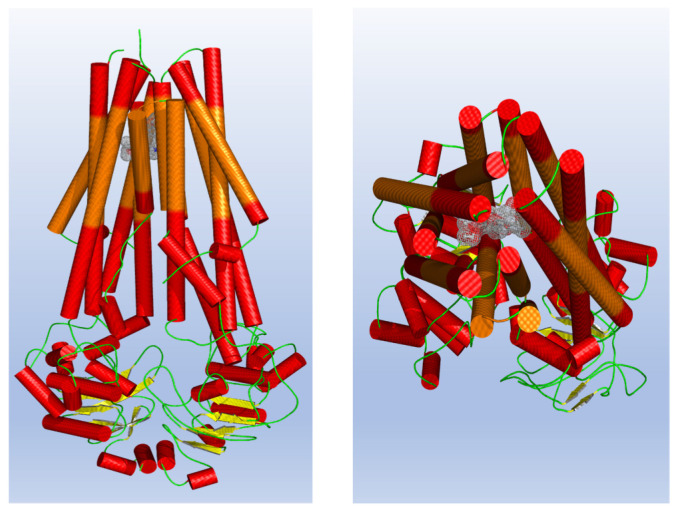
Front (**left**) and extracellular view (**right**) of the binding mode of **7c** to CryoEM P-gp structure. The transmembrane-spanning helices are depicted as orange, ligand in meshes.

Modeling studies help us to understand the probable binding mode to P-gp binding site(s) of the 1-Ph-DHPIQ-containing ligands and to rationalize their IC_50_ data. Molecular modeling studies on P-gp binders are now feasible thanks to many of the Cryo-EM solved structures. We felt confident to achieve deep insights into the DHPIQ ligands by docking to the so-called ‘inward-facing’ helix-binding site, hampering the ‘outward-facing’ shift of the protein, that is the basis of the transmembrane domains flipping leading to the pulling out of the xenobiotics. The binding modes of compounds **4a**, **4b**, **5c**, **6a**, **6c**, **7c**, **8b** and **8c** produced a sketch for a plausible interaction pattern of this type of ligand with the P-gp. As perceivable from Figure 3, the crevice located between the two six-helix domains typical of the class of ABCB1 binding cassette subfamily is largely occupied by the whole molecular scaffold of the inhibitors, which are then able to lock the in-out facing of the target protein.

**Figure 4:**
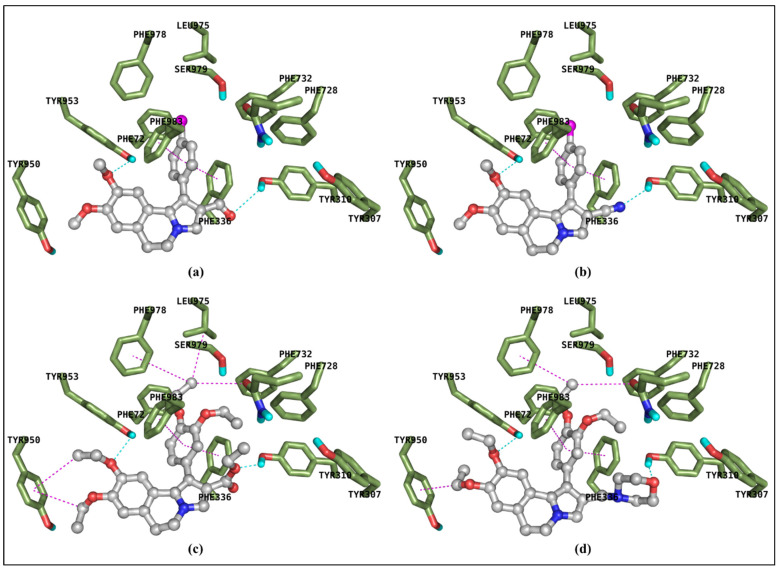
Detailed view of **4a** (**a**), **6a** (**b**), **7c** (**c**) and **8c** (**d**) highest scored docking modes. In the interaction pattern scheme, hydrogen bonds are depicted in cyan, Van der Waals contacts and π–π stackings in magenta.

Having proved this instance, additional insights were gained from ligand–residues interaction analysis of four DHPIQ compounds taken to be representative of the whole set, namely **4a** and **6a**, with IC_50_s in the low micromolar range, and the more active **7c** and **8c**, with sub-micromolar IC_50_s against P-gp (Figure 4). In the highest-scored docking modes of both **7c** (Figure 4c) and **8c** (Figure 4d), it appears that the diethoxy-phenyl moieties attain efficient π–π stacking with Phe336 and Phe983, at the same time engaging van der Waals contacts with Phe732, Phe978 and Leu975. Similar contacts may be attained by the two OEt substituents close to Tyr950. It is worth noting the hydrogen bonds (HBs) involving side chains of Tyr310 and Tyr95, which should assist the binding of both **7c** and **8c**.

The less potent 1-Ph-DHPIQ-based P-gp inhibitors **4a** (Figure 4a) and **6a** (Figure 4b), while similarly to **7c** and **8c** may attain a good π–π stacking between 4′-Cl-phenyl group and the aromatic side chains of Phe336 and Phe983, lose additional van der Waals contacts with Phe732, Phe978, Leu975, and even with Tyr950. A weaker HB between the aldehyde O (**4a**) or nitrile N (**6a**) and the phenol OH of Tyr310 may further explain the lower inhibitory potency of **4a** and **6a** compared with **7c** and **8c**.

In Table 3, the main docking calculation metrics are summarized for the representative compounds shown in Figure 4. As an additional figure of merit, a biphasic (quadratic) trend of correlation between pIC_50_ and the estimated free energy of binding (FEB, kcal·mol^−1^) was observed considering the docking models of all the inhibitors (Figure S10 in the Supplementary Materials).

## 3. Materials and Methods

### 3.1. Chemistry

All reagents and solvents were purchased from Merck (Darmstadt, Germany), J.T. Baker (Phillipsburg, NJ, USA), or Sigma-Aldrich Chemical Co. (St. Louis, MO, USA) and, unless specified, used without further purification. The melting points (m.p.) of all the compounds were determined on a SMELTING POINT 10 apparatus in open capillaries (Bibby Sterilin Ltd., Stone, UK). IR spectra were recorded on an Infralum FT-801 FTIR spectrometer (ISP SB RAS, Novosibirsk, Russia).Samples were analyzed as solid KBr disks, and the most important frequencies are expressed in cm^−1^. ^1^H and ^13^C NMR spectra were recorded in chloroform-*d* (CDCl_3_) or dimethylsulfoxide-*d_6_* (DMSO-d_6_) solutions at 25 °C, with a 600 MHz NMR spectrometer (JEOL Ltd., Tokyo, Japan). Peak positions are given in parts per million (ppm, *δ*) referenced to the appropriate solvent residual peak, and signal multiplicities are collected as: s (singlet), d (doublet), t (triplet), q (quartet), dd (doublet of doublets), br.s. (broad singlet) and m (multiplet). Mass spectra were recorded with an LCMS-8040 Triple quadrupole liquid chromatograph-mass spectrometer from Shimadzu (Kyoto, Japan). Elemental analyses were carried out on a Euro Vector EA-3000 Elemental Analyzer (Eurovector S.p.A., Milan, Italy) for C, H and N; experimental data agreed to within 0.4% of the theoretical values.

The synthetic procedures of compounds **4a**, **4c**–**e** [14] and **6a**,**c** [15] were recently described. Compounds **4b**, **5a**,**c**, **6d**,**e**, **7c**, **8b**,**c** and **8c**·**HCl** were synthesized according to the following procedures.

#### 3.1.1. Synthesis of (1-(4-Methoxyphenyl)-8,9-dimethoxy-5,6-dihydropyrrolo[2,1-*a*] Isoquinoline-2-carbaldehyde (**4b**)

Acrolein (118 mg, 2.10 mmol) was added to a solution of (6,7-dimethoxy-3,4-dihydroisoquinolin-1-yl)(4-methoxyphenyl)methanone (400 mg, 1.22 mmol) in trifluoroethanol (10 mL). The reaction was stirred at 40 °C for three hours. The reaction progress was monitored by TLC (sorbfil, EtOAc/hexane, 1:2). Then the solvent was removed under vacuum; the residue was crystallized from diethyl ether to afford compound **4b** as a beige powder (363 mg, 82%): mp 132–133 °C; ^1^H NMR (600 MHz, CDCl_3_,): *δ* = 3.04 (t, 2H, *J* = 6.3 Hz, 6-CH_2_), 3.38 (s, 3H, OCH_3_), 3.83 (s, 3H, OCH_3_), 3.85 (s, 3H, OCH_3_), 4.11 (t, 2H, *J* = 6.3 Hz, 5-CH_2_), 6.59 (s, 1H, 7-H), 6.68 (s, 1H, 10-H), 6.97 (d, 2H, *J* = 8.6 Hz, H-Ar), 7.35 (d, 2H, *J* = 8.6 Hz, H-Ar), 7.38 (s, 1H, 3-H), 9.62 (s, 1H, CHO); ^13^C NMR (150 MHz, CDCl_3_) *δ*: 29.1, 45.3, 55.2, 55.3, 55.9, 107.6, 111.1, 113.9 (2C), 121.2, 121.5, 124.2, 124.2, 125.0, 126.2, 127.2, 131.9 (2C), 147.6, 147.6, 159.0, 186.5; MS (LCMS) *m*/*z* = 364 [M+H]^+^; anal. calcd. for C_22_H_21_NO_4_ (%): C 72.71, H 5.82, N 3.85, found: C 72.83, H 5.63, N 3.93.

#### 3.1.2. Synthesis of 1-Aryl-5,6-dihydropyrrolo[2,1-*a*]isoquinoline-2-carboxylic acids **5a**,**c**

We added 4-nitrophenylacrylate (1.20 mmol) to a solution of corresponding 1-aroylisoquinolines (1.0 mmol) in trifluoroethanol (5 mL). The reaction proceeded under microwave activation for 1 h at 140 °C. The progress of the reaction was monitored by TLC (sorbfil, EtOAc/hexane, 1:1). The solvent was evaporated, the residue was crystallized from ether.

1-(4-Chlorophenyl)-8,9-dimethoxy-5,6-dihydropyrrolo[2,1-*a*]isoquinoline-2-carboxylic acid (**5a**): Yellow powder (345 mg, 90%): mp 210–215 °C; ^1^H NMR (600 MHz, CDCl_3_): *δ* = 3.03 (t, 2H, *J* = 6.6 Hz, 6-CH_2_), 3.34 (s, 3H, OCH_3_), 3.84 (s, 3H, OCH_3_), 4.10 (t, 2H, *J* = 6.6 Hz, 5-CH_2_), 6.36 (s, 1H, 7-H), 6.67 (s, 1H, 10-H), 7.33 (d, 2H, *J* = 8.6 Hz, H-Ar), 7.38 (d, 2H, *J* = 8.6 Hz, H-Ar), 7.44 (s, 1H, 3-H); ^13^C NMR (150 MHz, CDCl_3_): *δ* = 29.2, 45.2, 55.2, 56.0, 107.7, 111.2, 112.3, 120.3, 120.9, 124.3, 126.7, 127.8, 128.5 (2C), 132.2 (2C), 133.2, 134.1, 147.7, 147.8, 162.4. MS (LCMS) *m*/*z* = 384 [M+H]^+^; anal. calcd for C_21_H_18_ClNO_4_, (%): C, 65.71; H, 4.73; N, 3.65, found: C, 65.98; H, 4.94; N, 3.83.

1-(3,4-Diethoxyphenyl)-8,9-diethoxy-5,6-dihydropyrrolo[2,1-*a*]isoquinoline-2-carboxylic acid (**5c**): Beige powder (372 mg, 80%): mp 170–172 °C; ^1^H NMR (600 MHz, CDCl_3_): *δ* = 1.16 (t, 3H, *J* = 7.1 Hz, OCH_2_CH_3_), 1.37–1.42 (m, 6H, OCH_2_CH_3_), 1.45 (t, 3H, *J* = 7.1 Hz, OCH_2_CH_3_), 3.00 (t, 2H, *J* = 6.6 Hz, 6-CH_2_), 3.56 (q, 2H, *J* = 7.1 Hz, OCH_2_CH_3_), 4.01–4.06 (m, 4H, OCH_2_CH_3_), 4.09 (t, 2H, *J* = 6.6 Hz, 5-CH_2_), 4.11 (q, 2H, *J* = 7.1 Hz, OCH_2_CH_3_), 6.53 (s, 1H, 7-H), 6.66 (s, 1H, 10-H), 6.88–6.92 (m, 3H, H-Ar), 7.41 (s, 1H, 3-H); ^13^C NMR (150 MHz, CDCl_3_): *δ* = 14.6, 14.9 (2C), 15.0, 29.2, 45.3, 63.8, 64.5, 64.7, 64.8, 109.3, 112.5, 113.3, 113.8, 116.1, 121.4, 121.5, 123.0, 123.9, 126.4, 127.7, 128.3, 147.2, 147.3, 148.0, 148.9, 162.5; MS (LCMS) *m*/*z* = 466 [M+H]^+^; anal. calcd for C_27_H_31_NO_6_, (%): C, 69.66; H, 6.71; N, 3.01, found: C, 70.23; H, 6.88; N, 3.12.

#### 3.1.3. Synthesis of Carbonitriles **6d**,**e**

Nitriles **6d**,**e** were obtained in two stages. In the first step, compounds **4d** or **4e** (0.3 mmol) were dissolved in 7 mL of ethanol. Hydroxylamine hydrochloride (2.0 mmol) and sodium acetate (3.0 mmol) were added to the resulting solution, and the mixtures were refluxed for 14 h. The reaction progress was monitored by TLC (sorbfil, EtOAc/hexane, 1:2). The solvent was removed under vacuum; the residue was crystallized from ether to produce oximes as a beige powder. After filtration and drying in the second stage, the obtained oximes were boiled in 3 mL of acetic anhydride until the initial spot disappeared on TLC. Ice was added to the mixture, sodium bicarbonate was added to pH 8, and the mixture was extracted with EtOAc (3 × 10 mL). After removing the solvent, the residue was recrystallized from EtOAc/hexane.

1-(3,4-Diethoxyphenyl)-8,9-diethoxy-3-methyl-5,6-dihydropyrrolo[2,1-*a*]isoquinoline-2-carbonitrile (**6d**): Beige powder (46 mg, 50%): mp 270–272 °C; ^1^H NMR (600 MHz, CDCl_3_): δ = 1.20 (t, 3H, *J* = 7.1 Hz, OCH_2_CH_3_), 1.40–1.46 (m, 9H, OCH_2_CH_3_), 2.42 (s, 3H, CH_3_), 2.99 (t, 2H, *J* = 6.6 Hz, 6-CH_2_), 3.61 (q, 2H, *J* = 7.1 Hz, OCH_2_CH_3_), 3.92 (t, 2H, *J* = 6.6 Hz, 5-CH_2_), 4.04–4.08 (m, 4H, OCH_2_CH_3_), 4.12 (q, 2H, *J* = 7.1 Hz, OCH_2_CH_3_), 6.68 (s, 1H, 7-H), 6.80 (s, 1H, 10-H), 6.90 (d, 1H, *J* = 8.6 Hz, H-Ar), 6.98 (br.s, 2H, H-Ar); ^13^C NMR (150 MHz, CDCl3): δ = 11.3, 14.7 (2C), 14.9 (2C), 29.0, 41.8, 64.1, 64.6, 64.7, 64.8, 93.5, 109.4, 113.1, 114.0, 114.9, 117.2, 121.2, 121.4, 122.2 (2C), 123.9, 125.3, 126.7, 136.2, 147.5, 148.1, 149.0; MS (LCMS) *m*/*z* = 461 [M+H]^+^; anal. calcd for C_28_H_32_N_2_O_4_, (%): C, 73.02; H, 7.00; N, 6.08, found: C, 72.98; H, 6.93; N, 5.81.

1-(3,4-Diethoxyphenyl)-8,9-diethoxy-3-phenyl-5,6-dihydropyrrolo [2,1-*a*]isoquinoline-2-carbonitrile (**6e**): Brown powder (68 mg, 65%): mp 271–273 °C; ^1^H NMR (600 MHz, CDCl_3_): *δ* = 1.22 (t, 3H, *J* = 7.1 Hz, OCH_2_CH_3_), 1.41–1.47 (m, 9H, OCH_2_CH_3_), 2.95 (t, 2H, *J* = 6.6 Hz, 6-CH_2_), 3.64 (q, 2H, *J* = 7.1 Hz, OCH_2_CH_3_), 4.06 (t, 2H, *J* = 6.6 Hz, 5-CH_2_), 4.07 (q, 4H, *J* = 7.1 Hz, OCH_2_CH_3_), 4.14 (q, 2H, *J* = 7.1 Hz, OCH_2_CH_3_), 6.70 (s, 1H, 7-H), 6.84 (s, 1H, 10-H), 6.94 (d, 1H, *J* = 8.6 Hz, H-Ar), 7.04 (d, 2H, *J* = 6.1 Hz, H-Ar), 7.43–7.45 (m, 1H, H-Ar), 7.49–7.51 (m, 4H, H-Ar); ^13^C NMR (150 MHz, CDCl_3_): *δ* = 14.7, 14.9 (2C), 29.3, 43.2, 64.1, 64.6, 64.7, 64.8, 94.2, 109.9, 112.9, 114.0, 115.1, 117.2, 121.0, 122.4 (2C), 122.9, 124.9, 126.3, 126.5, 128.9 (2C), 129.0, 129.1, 129.6 (2C), 139.3, 147.4, 147.7, 148.3, 149.1; MS (LCMS) *m*/*z* = 523 [M+H]^+^; anal. calcd for C_33_H_34_N_2_O_4_, (%): C, 75.84; H, 6.56; N, 5.36, found: C, 75.91; H, 6.86; N, 5.42.

#### 3.1.4. Synthesis of Ethyl 1-(3,4-Diethoxyphenyl)-8,9-diethoxy-5,6-dihydro pyrrolo[2,1-*a*]isoquinoline-2-carboxylate (**7c**)

Ethyl acrylate (150 mg, 1.5 mmol) and ZnO (8 mg, 10 mol%) were added to a solution of (6,7-diethoxy-3,4-dihydroisoquinolin-1-yl)(3,4-diethoxyphenyl)methanone (411 mg, 1.0 mmol) in trifluoroethanol (10 mL). The reaction was carried out at 140 °C for 30 min. The progress of the reaction was monitored by TLC (sorbfil, EtOAc/hexane, 1:1). The solvent was removed; the residue was treated with sodium acetate solution and extracted with EtOAc (3 × 10 mL). After removal of the solvent, 5 mL of toluene was added and distilled to dryness to remove unreacted ethyl acrylate. The residue was crystallized from ether. White powder (296 mg, 60%): mp 170–172 °C; ^1^H NMR (600 MHz, CDCl_3_): *δ* = 1.12 (t, 3H, *J* = 7.1 Hz, OCH_2_CH_3_), 1.15 (t, 3H, *J* = 6.1 Hz, OCH_2_CH_3_), 1.37–1.42 (m, 6H, OCH_2_CH_3_), 1.45 (t, 3H, *J* = 7.1 Hz, OCH_2_CH_3_), 2.98 (t, 2H, *J* = 6.6 Hz, 6-CH_2_), 3.56 (q, 2H, *J* = 7.1 Hz, OCH_2_CH_3_), 4.03 (q, 2H, *J* = 7.1 Hz, OCH_2_CH_3_), 4.05–4.08 (m, 4H, 5-CH_2_, OCH_2_CH_3_), 4.11 (q, 4H, *J* = 7.1 Hz, OCH_2_CH_3_), 6.53 (s, 1H, 7-H), 6.56 (s, 1H, 10-H), 6.88–6.90 (m, 3H, H-Ar), 7.34 (s, 1H, 3-H); ^13^C NMR (150 MHz, CDCl_3_): *δ* = 14.2, 14.8 (2C), 14.9 (2C), 28.1, 43.7, 60.2, 64.9, 65.7, 65.8, 65.9, 105.6, 112.0, 113.2, 113.9, 116.5, 120.4, 120.7, 126.1, 126.9, 129.2, 132.4, 132.5, 146.7, 148.9, 149.0, 152.1, 161.9; MS (LCMS) *m*/*z* = 494 [M+H]^+^; anal. calcd for C_29_H_35_NO_6_, (%): C, 70.57; H, 7.15; N, 2.84, found: C, 70.64; H, 6.95; N, 3.12.

#### 3.1.5. Synthesis of 2-(Morpholin-4-yl-methyl)-5,6-dihydropyrrolo[2,1-*a*]isoquinolines **8b**,**c**

Morpholine (1.34 mmol) was added to a solution of pyrrolo[2,1-*a*]isoquinoline-2-carbaldehydes **4b,c** (0.67 mmol) in acetonitrile (15 mL). The mixture was boiled for 20 h. Then acetonitrile was replaced by methanol, sodium borohydride (2.68 mmol) was added in portions, and the mixture was boiled for 1 h. The reaction mass was then cooled, and the solvent was evaporated. A saturated sodium bicarbonate solution (30 mL) was added to the dry residue and extracted with EtOAc (3 × 15 mL). The solvent was evaporated, compound **8b** was isolated as oils, and compound **8c** was crystallized from a mixture of diethyl ether and EtOAc.

8,9-Dimethoxy-1-(4-methoxyphenyl)-2-(morpholin-4-ylmethyl)-5,6-dihydropyrrolo[2,1-*a*]isoquinoline (**8b**): Yellow oil (154 mg, 53%); ^1^H NMR (600 MHz, CDCl_3_): δ = 2.36–2.45 (m, 4H, CH_2_ (morpholinyl)), 2.99 (t, 2H, *J* = 6.6 Hz, 6-CH_2_), 3.27 (s, 2H, CH_2_N), 3.36 (s, 3H, OCH_3_), 3.67 (t, 4H, *J* = 4.5 Hz, CH_2_ (morpholinyl)), 3.82 (s, 3H, OCH_3_), 3.83 (s, 3H, OCH_3_), 4.01 (t, 2H, *J* = 6.6 Hz, 5-CH_2_), 6.59 (s, 1H, 7-H), 6.64 (s, 1H, 10-H), 6.65 (s, 1H, 3-H), 6.92 (d, 2H, *J* = 8.6 Hz, H-Ar), 7.36 (d, 2H, *J* = 8.6 Hz, H-Ar); ^13^C NMR (150 MHz, CDCl_3_): δ = 29.3, 42.1, 47.8, 53.1 (2C), 54.5, 55.1, 55.6, 66.5, 66.6, 105.5, 111.4, 111.7, 112.8, 117.4, 122.8, 123.2, 123.9, 124.4, 125.4 (2C), 127.9, 132.3, 148.0, 148.9, 160.2; MS (LCMS) *m*/*z* = 435 [M+H]^+^; anal. calcd for C_26_H_30_N_2_O_4_, (%): C, 71.87; H, 6.96; N 6.45, found: C, 71.95; H, 7.03; N, 6.54.

1-(3,4-Diethoxyphenyl)-8,9-diethoxy-2-(morpholin-4-ylmethyl)-5,6-dihydropyrrolo[2,1-*a*]isoquinoline (**8c**): White powder (223 mg, 64%): mp 72–73 °C; ^1^H NMR (600 MHz, CDCl_3_): δ = 1.17 (t, 3H, *J* = 7.1 Hz, OCH_2_CH_3_), 1.40–1.43 (m, 6H, OCH_2_CH_3_), 1.46 (t, 3H, *J* = 7.1 Hz, OCH_2_CH_3_), 2.35–2.45 (m, 4H, CH_2_ (morpholinyl), 2.97 (t, 2H, *J* = 6.2 Hz, 6-CH_2_), 3.26 (br.s., 2H, CH_2_N), 3.58 (q, 2H, *J* = 7.1 Hz, OCH_2_CH_3_), 3.64–3.67 (m, 4H, CH_2_ (morpholinyl), 3.99 (t, 2H, *J* = 6.2 Hz, 5-CH_2_), 4.02–4.08 (m, 4H, OCH_2_CH_3_), 4.11 (q, 2H, *J* = 7.1 Hz, OCH_2_CH_3_), 6.62 (s, 1H, 7-H), 6.65 (s, 1H, 10-H), 6.67 (s, 1H, H-Ar), 6.88 (d, 1H, *J* = 8.1 Hz, H-Ar), 6.92 (dd, 1H, *J* = 1.5, 8.1 Hz, H-Ar), 7.07 (s, 1H, 3-H); ^13^C NMR (150 MHz, CDCl3): δ = 14.5, 15.2 (2C), 15.4, 29.2, 42.1, 46.9, 53.1, 53.2, 64.9, 65.0, 65.1, 65.8, 66.5 (2C), 110.1, 110.7, 113.3, 113.7, 117.0, 118.1, 123.1, 123.7, 124.4, 125.3, 125.9, 133.1, 148.5, 148.9, 149.0, 150.7; MS (LCMS) *m*/*z* = 521 [M+H]^+^. Anal. calcd for C_31_H_40_N_2_O_5_, (%): C, 71.51; H, 7.74; N, 5.38, found: C, 71.33; H, 7.92; N, 5.21.

#### 3.1.6. Synthesis of 4-{[1-(3,4-Diethoxyphenyl)-8,9-diethoxy-5,6-dihydropyrrolo[2,1-*a*]isoquinolin-2-yl]methyl}morpholin-4-ium chloride (**8c**·**HCl**)

Concentrated hydrochloric acid was added dropwise to a solution of compound **8b** (52 mg, 0.10 mmol) in DCM (2 mL) until pH 2. The solvent was removed and the residue was crystallized with ether to afford compound **8c**·**HCl** as a white powder (49 mg, 87%): mp 90–92 °C; ^1^H NMR (600 MHz, DMSO-d_6_): *δ* = 1.14 (t, 3H, *J* = 7.1 Hz, OCH_2_CH_3_), 1.40–1.43 (m, 6H, OCH_2_CH_3_), 1.48 (t, 3H, *J* = 7.1 Hz, OCH_2_CH_3_), 1.77–1.80 (m, 2H, CH_2_ (morpholinyl)), 2.51–2.52 (m, 2H, CH_2_ (morpholinyl)), 2.98 (br.s., 2H, CH_2_N), 3.17–3.20 (m, 2H, CH_2_ (morpholinyl)), 3.56 (q, 2H, *J* = 7.1 Hz, OCH_2_CH_3_), 3.78–3.82 (m, 2H, 6-CH_2_), 3.98–4.07 (m, 8H, OCH_2_CH_3_, 5-CH_2_, CH_2_ (morpholinyl)), 4.11 (q, 2H, *J* = 7.1 Hz, OCH_2_CH_3_), 6.51 (s, 1H, 7-H), 6.66 (s, 1H, 10-H), 6.76–6.78 (m, 2H, H-Ar), 6.93 (d, 1H, *J* = 8.1 Hz, H-Ar), 7.38 (s, 1H, 3-H), 12.41 (br.s., 1H, NH^+^ (morpholinyl)); ^13^C NMR (150 MHz, DMSO-d_6_): *δ* = 14.6 (2C), 14.9 (2C), 15.0, 29.2 (2C), 44.9 (2C), 51.9, 63.7, 63.9, 64.7, 64.8 (2C), 108.8, 109.0, 113.4, 114.1, 118.0, 121.1, 121.5, 123.2, 123.4, 124.1, 126.3, 127.8, 147.1, 147.2, 148.3, 149.4; MS (LCMS) *m*/*z* = 521 [M-Cl]^+^; anal. calcd for C_31_H_40_N_2_O_5_ × HCl, (%): C, 66.83; H, 7.42; N, 5.03, found: C, 67.03; H, 7.55; N, 5.21.

### 3.2. Biological Evaluation

#### 3.2.1. Cell Cultures

Human cell cultures RD (rhabdomyosarcoma, ATCC CCL-136), HCT116 (intestinal carcinoma, ATCC CCL-247), HeLa (cervical adenocarcinoma, ATCC CCL-2) and A549 (lung carcinoma, ATCC CCL-185), obtained from the Institute of Cytology RAS, Saint Petersburg (Russian Collection of Cell Cultures of Vertebrates), were grown in DMEM (for RD, HCT116 and A549) and EMEM (for HeLa) supplemented with 10% fetal calf serum, 2 mM l-glutamine and 1% gentamicin as an antibiotic at 37 °C and 5% CO_2_ in a humid atmosphere. MDCK-MDR1 and MDCK-MRP1 were a gift from Prof. P. Borst, NKI-AVL Institute, Amsterdam, The Netherlands. MDCK-MDR1 and MDCK-MRP1 were grown in high-glucose DMEM supplemented with 10% fetal bovine serum, l-glutamine (2 mM), penicillin (100 U mL^−1^) and streptomycin (100 mg mL^−1^) in a humidified incubator at 37 °C in a 5% CO_2_ atmosphere.

#### 3.2.2. Cytotoxicity Assay

The antiproliferative activity of the newly synthesized compounds, along with some previously reported analogs, and camptothecin and doxorubicin as positive controls was determined by the MTT (3-(4,5-dimethylthiazol-2-yl)-2,5-diphenyltetrazolium bromide) assay. The cells were seeded at a concentration of 1 × 10 ^4^ cells/200 μL in a 96-well plate and cultured at 37 °C in a humidified atmosphere with 5% CO_2_. After 24 h of incubation, scalar concentrations of each test compound (100 to 1.56 μmol·L^−1^) were added to the cancer cell culture and the cells were then cultured under the same conditions for 72 hr. Each concentration of the test compound was assayed in triplicate. All substances were dissolved in DMSO, whose final concentration in the well did not exceed 0.1% *v*/*v* and proved to be not toxic to the cells. Wells with the 0.1% *v*/*v* DMSO were used as controls. After incubation, 20 μL of MTT (5 mg·mL^−1^) was added to each well and the plates were incubated for a further 2 h. Next, media were removed from the plates, and 100 μL of DMSO was added to each well to dissolve the formed formazan crystals. Using a flatbed analyzer (Victor^3^, PerkinElmer, Waltham, MA, USA), the optical density was determined at 530 nm, minus the measured background absorbance at 620 nm. The concentration value, which causes 50% inhibition of cell population growth (IC_50_), was determined from the dose-dependent curves using the OriginPro 9.0 software.

#### 3.2.3. Inhibition Assays of P-Glycoprotein (P-gp) and Multidrug-Resistance-Associated Protein-1 (MRP1)

According to previously described assays [9], MDCK-MDR1 and MDCK-MRP1 cell lines (50,000 cells per well) were seeded into a black CulturePlate 96-well plate with 100 μL medium and allowed to become confluent overnight. Then, 100 μL of scalar concentrations of each test compound (100 to 0.1 μM) was solubilized in the culture medium and added to each well. The 96-well plate was incubated at 37 °C for 30 min, and 100 μL of Calcein-AM in PBS was added to each well to yield a final concentration of 2.5 mM; the plate was incubated for 30 min and then washed three times with 100 mL ice-cold PBS. Saline buffer (100 μL) was added to each well, and the plate was read by a PerkinElmer Victor3 spectrofluorimeter at excitation and emission wavelengths of 485 nm and 535 nm, respectively. In these experimental conditions, calcein cell accumulation in the absence and in the presence of the test compound was evaluated, and the fluorescence basal level was estimated by untreated cells. In the treated wells, the increase of fluorescence with respect to basal level was measured. IC_50_ values were determined by interpolating the curve of fluorescence increase percentage versus log [conc.].

#### 3.2.4. Affinity to Human Serum Albumin (HSA) by Surface Plasmon Resonance (SPR)

Fatty acid-free HSA (A3782 from Merck, KGaA Sigma-Aldrich, Darmstadt, Germany) was applied to functionalize the surface of a COOH V sensor chip (Pall FortèBio, Fremont Boulevard, CA, USA), by using amine coupling (EDC/NHS). HSA aqueous stock solution was diluted at the final concentration of 50 μg·mL^−1^ in 10 mM sodium acetate buffer (pH 5.0), and immobilization in peripheral flow cells 1 and 3 (unmodified dextran surface in middle flow cell 2 used as a reference) at a final apparent level of about 5000 RU was achieved by applying the following protocol: injection of EDC/NHS (freshly mixed 0.4 M EDC and 0.1 M NHS) 1:1 *v*/*v* at a flow of 25 μL·min^−1^ for 4 min; injection of HSA solution at a flow of 25 μL·min^−1^ for 8 min; capping on unreacted activated carboxyl groups by injection of 1 M ethanolamine solution (pH 9) at a flow of 25 μL·min^−1^ for 8 min. The functionalized surface was then treated with repeated injection of 4 M NaCl, as a regenerating reagent, and conditioned overnight prior to use with pH 7.4 PBS (10 mM NaH_2_PO_4_ and 150 mM NaCl) as a running buffer. Each cycle (without the need of regeneration) for binding assay to HSA was carried out in PBS/4% *v*/*v* DMSO and consisted of a run buffer injection (60 s), injection of analyte single concentration for 120 s (ranging from 1 to 200 μM) at a flow 20 μL·min^−1^, and a dissociation phase for 150 s (flow 20 μL·min^−1^). Each measurement was performed at least in triplicate and analyzed by using QDAT software, vers 2.2.0.7 (non-linear regression analysis of 1:1 stoichiometric reversible binding model), and double referencing.

### 3.3. Aqueous Solubility and Lipophilicity

#### 3.3.1. Determination of Kinetic Solubility in PBS

Sample solution at 200 μM in PBS (50 mM, pH 7.4, KCl 0.15 M) from a 10 mM stock solution in DMSO was incubated at room temperature (25 ± 1 °C) for 2 h, following shaking of the suspension on an orbital shaker at 250 rpm, and then separated by centrifugation (2500 rpm for 3 min). Immediately after the filtration step, 100 µL of filtrate was transferred into 100 µL of 1:1 (*v*/*v*) mixture of DMSO and PBS (50 mM, pH 7.4, KCl 0.15 M), to avoid precipitation from the saturated solution and analyzed by HPLC. The peak area was plotted against a calibration curve of the tested compound in MeOH [21]. Analytical conditions were as follows. Mobile Phase: MeOH/ammonium formate 20 mM pH 4.7 (70:30, 75:25); MeOH 0.1% TFA/H_2_O 0.1% TFA (70:30, 60:40); stationary phase: Phenomenex, Kinetex 5 μ, C18, 100 Å (150 × 4.6 mm); flux: 1 mL/min; injection: 10 μL, 265, 290, 320 nm wavelength. HPLC analyses were performed on an Agilent HPLC 1260 Infinity Series Integrated System (Agilent Technologies, Milan, Italy).

#### 3.3.2. Determination of Lipophilicity by RP-HPLC

The lipophilicity parameters were determined by an RP-HPLC technique [20]. Methanol solution of DHPIQ derivatives (1 mg/mL) were analyzed by an Agilent 1260 infinite HPLC system (Agilent Technologies, Milan, Italy) equipped with a diode array detector (DAD), and a Phenomenex, Kinetex 5 μ, C18, 100 Å (150 × 4.6 mm), and eluted with different percentage of mobile phase composition (0.05 increments of MeOH volume fractions in 20 mM ammonium formate buffer at pH 4.7 or water 0.1% *v*/*v* TFA, with φ ranging between 0.85 and 0.30). The chromatographic measurements were carried out at 25 ± 1 °C at a flow rate of 1 mL·min^−1^ and at 265, 290, 320 nm wavelengths. The log of capacity factors (log *k*′ = log (t_R_ − t_0_)/t_0_) of each compound at different mobile phase compositions was calculated; t_R_ represents the retention time of the solute and t_0_ is the column dead time, measured as the elution time of a KNO_3_ solution in MeOH. For each compound, the log *k*′ values increased linearly with decreasing MeOH volume fraction. The logarithms of the capacity factor extrapolated to 100% aqueous mobile phase (log *k*′_w_) were calculated from the linear regressions on at least five data points (*r*^2^ > 0.9984).

Lipophilicity was also computationally assessed as log *P* and log *D* values at pH 4.7 (pH of the aqueous buffer in the mobile phase for RP-HPLC) using ACDLabs software (release 10.0; Advanced Chemistry Development, Inc., Toronto, ON, Canada). The values of calculated log P (Clog P), referred to lipophilicity of the neutral species, are listed in Table 2 together with the RP-HPLC parameters log *k′*_w_. Log *k′*_w_ and Clog P were reasonably correlated (*r*^2^ = 0.7911), but an inspection of the correlation plot suggested that, omitting from the regression analysis the two 2-morpholinomethyl DHPIQ derivatives **8b** and **8c**, which should be predominantly protonated (positively charged) at the tertiary amino group, all the other data points fit well the following linear equation:

Log *k′*_w_ = 0.78 (±0.16) Clog P − 0.12 (±0.99)

*n* = 6, *r*^2^ = 0.8592, *s* = 0.3699, *F* = 24.41

where *n* is the number of data points, *r*^2^ is the coefficient of determination, *s* is the standard deviation of the regression equation, and *F* is the F-value from the Fisher test for regression model significance (95% confidence intervals of the regression coefficients are given in parentheses).

### 3.4. Molecular Dooking Calculations

The ligands’ structures were built starting from the relative SMILES strings converted to three-dimensional structures with OMEGA [22]; following this, 10,000 steps of steepest descent minimization using the UFF was performed with Open Babel (vers 3.1.0) [23]. The target molecule (P-gp) was prepared starting from the MDR1 Cryo-EM structure (Protein Data Bank entry 7A6C) [24] with the Protein Preparation Wizard interface of Maestro [25], removing the co-crystallized elacridar molecule, loading and optimizing hydrogen atom positions, and assigning the ionization states of acidic and basic residues according to PROPKA prediction at pH 7.0. Electrostatic charges for protein atoms were loaded according to the AMBER UNITED force field [26], while the *molcharge* set of QUACPAC (vers 2.2.0.4) [27] was used to achieve Marsili–Gasteiger charges for the inhibitors.

Solvent was explicitly considered by means of the proper parametrization of water contribution according to the relative AUTODOCK hydration force field [28], and the population size and the number of energy evaluation figures were set to 300 and 10,000,000, respectively. Dockings were then performed throughout 1000 runs of the Lamarckian genetic algorithm (LGA) implemented in AUTODOCK 4.2.6 [29] using the GPU-OpenCL algorithm version [30], and the best energy/best cluster poses as scored by AUTODOCK were selected.

## 4. Conclusions

In our recent studies on the annelated azaheterocyclic core of the marine alkaloids lamellarins [5,8,9], we disclosed novel DHPIQ carbaldehydes (and related imino adducts) showing anticancer activities, in some cases coupled with inhibition of the P-gp efflux pump [9]. While in-depth studies are needed to understand their (likely multitarget) mechanism of action, herein, using a classical bioisosteric replacement approach, we investigated the role of the electrophilic 2-CHO group in modulating the antiproliferative activity in tumor cells and P-gp/MRP1 inhibition potency. The aldehyde group CHO was replaced by the less reactive electrophilic nitrile CN [10], as well as by the carboxylic group in 2-COOH and 2-COOEt derivatives. The replacement of CHO with CN, COOH and COOEt resulted in a sharp decrease of cytotoxic activity against all four tumor cell lines tested (RD, HCT116, HeLa, A549). The comparison between the cytotoxicities of the 2-CHO derivatives (**4a** and **4c**) and the corresponding 2-CN analogs (**6a** and **6c**), although they possess almost similar stereoelectronic and lipophilic features, suggests that CHO, and not CN, may be involved in covalent reactions with nucleophiles of biological targets (DNA, enzyme proteins). The lipophilicity of the substituents onto the 1-Ph-DHPIQ scaffold appeared to play a secondary role in affecting cytotoxicity, whereas in contrast, regardless of the main functional group in the C2 position (CHO, CN, COOH, COOEt), as a trend, the more lipophilic, the more potent the inhibitor of P-gp (and not MRP1). The P-gp inhibition SARs were usefully supported by in silico docking calculation models.

Unfortunately, except the ester **7c**, all of the above derivatives (**4**, **5** and **6**) were very poorly soluble in aqueous solutions at physiological pHs. A successful attempt pursued in this study to improve the water solubility was the synthesis of a couple of DHPIQs bearing at C2 the basic 2-morpholinomethyl chain, which was estimated to be more than half in protonated form at neutral pH. Interestingly, the novel DHPIQ Mannich base **8c**, prepared as a HCl salt, stable at pHs 2 and 7.4 at room temperature, proved to be cytotoxic to all of the tested tumor cell lines in the low micromolar range (IC_50_ < 20 μM) and to inhibit in vitro the efflux pumps P-gp and MRP1 responsible for MDR, with IC_50_ of 0.45 and 12.1 μM, respectively. The basic compounds **8b** and **8c** were significantly (three- to five-fold) more soluble than the related aldehydes, but still below the minimum solubility threshold of 0.1 mg·mL^−1^ according to the categorization of the US and Eur. Pharmacopoeia [19]. Nevertheless, the synthesis of DHPIQs **8a** and **8c** provided a useful approach to prepare new water-soluble lamellarin-like antineoplastic substances, including more hydrophilic prodrugs and Mannich bases, endowed with the ability of overcoming P-gp-mediated MDR.

## Figures and Tables

**Table 1:** In vitro cytotoxicity data in human cancer cell cultures ^a^.

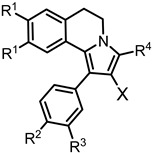									
N	X	R^1^	R^2^	R^3^	R^4^	RD	HCT116	HeLa	A549
**4a** ^b^	CHO	OMe	Cl	H	H	17.6 (3.2)	22.0 (4.0)	33.0 (4.5)	38.6 (3.2)
**4b**	CHO	OMe	OMe	H	H	95.2 (7.1)	>100	>100	>100
**4c** ^c^	CHO	OEt	OEt	OEt	H	21.3 (1.2)	11.8 (0.2)	44.5 (2.0)	19.7 (0.3)
**5a**	CO_2_H	OMe	Cl	H	H	>100	>100	>100	>100
**5c**	CO_2_H	OEt	OEt	OEt	H	n.a.	n.a.	n.a.	n.a.
**6a**	CN	OMe	Cl	H	H	>100	>100	>100	>100
**6c**	CN	OEt	OEt	OEt	H	>100	>100	>100	>100
**6d**	CN	OEt	OEt	OEt	Me	>100	>100	>100	>100
**6e**	CN	OEt	OEt	OEt	Ph	>100	n.a.	n.a.	n.a.
**7c**	CO_2_Et	OEt	OEt	OEt	H	n.a.	n.a.	n.a.	n.a.
**8b**	MM ^d^	OMe	OMe	H	H	37.7 (1.6)	56.4 (1.7)	65.4 (4.5)	66.0 (1.6)
**8c**	MM·HCl	OEt	OEt	OEt	H	18.6 (2.7)	15.7 (0.7)	17.5 (1.4)	20.8 (3.6)
Camptothecin						16.0 (0.2)	12.3 (0.5)	0.33 (0.07)	3.32 (0.02)
Doxorubicin						0.29 (0.02)	0.14 (0.01)	0.89 (0.01)	0.38 (0.02)

^a^ RD: rhabdomyosarcoma; HCT116: colon cancer cells; HeLa: adenocarcinoma of the cervix uterus; A549: lung adenocarcinoma. Each experiment was performed in triplicate; data are expressed as the mean µM IC_50_ (SD), that is, the concentration value causing 50% inhibition of cell population growth. ^b^ Ref. [5]. ^c^ Ref. [8]. ^d^ MM = morpholinomethyl. n.a.: not active at 100 μM; >100: less than 60% inhibition at 100 μM.

**Table 2:** Inhibitory potencies of 1-Ph-DHPIQ derivatives toward P-gp and MRP1 drug efflux pumps, and ADME-related physicochemical parameters.

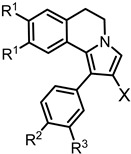										
N	X	R^1^	R^2^	R^3^	P-gp, IC_50_ (μM) ^a^	MRP1, IC_50_ (μM) ^a^	HSA, *K*_D_ (μM) ^b^	S (μM) ^c^	CLog P ^d^	Log *k′*_w_ ^e^
**4a**	CHO	OMe	Cl	H	25.3 (1.9)	21.9 (1.5)	1.60 (0.04)	6.66 (0.09)	5.62	4.32
**4b**	CHO	OMe	OMe	H	4.48 (0.17)	6.42 (0.27)	1.95 (0.06)	19.8 (0.1)	4.86	3.39
**5c**	CO_2_H	OEt	OEt	OEt	0.35 (0.04)	>100	5.20 (0.20)	14.6 (0.6)	4.70	6.91
**6a**	CN	OMe	Cl	H	5.89 (0.42)	16.6 (0.5)	4.60 (0.10)	1.42 (0.01)	5.75	4.42
**6c**	CN	OEt	OEt	OEt	0.39 (0.06)	>100	1.80 (0.20)	1.06 (0.05)	6.79	5.09
**7c**	CO_2_Et	OEt	OEt	OEt	0.32 (0.08)	3.23 (0.29)	12.1 (0.1)	41.7 (1.1) ^g^	7.75	5.49
**8b**	MM ^f^	OMe	OMe	H	0.36 (0.02)	1.80 (0.31)	19.3 (0.5)	112 (4) ^g^	4.73	3.86
**8c**	MM·HCl	OEt	OEt	OEt	0.45 (0.03)	12.1 (2.1)	26.9 (0.5)	41.7 (1.2) ^g^	6.53	4.31
*MC18*					1.20 (0.3)					
Verapamil						4.53 (0.50)				
Warfarin							5.30 (0.35)			

^a^ Inhibition potency data toward P-glycoprotein (P-gp) and multidrug-resistance-associated protein-1 (MRP1), expressed by mean IC_50_ (SD) values of at least two independent experiments each performed in triplicate; >100: less than 60% inhibition at 100 μM. MC18 and verapamil were assayed as the P-gp-selective and MRP1-selective positive controls, respectively. ^b^ Binding to human serum albumin (HSA) determined by surface plasmon resonance technique, expressed by at equilibrium dissociation constants (*K*_D_ values); the affinity of warfarin was reported as reference value. ^c^ Solubility of 1-Ph-DHIQ derivatives in PBS (50 mM, pH 7.4, 0.15 M KCl) at 25 ± 1 °C. ^d^ Log of 1-octanol/water partition coefficient calculated by ACDLabs software (release 10.0, Advanced Chemistry Development, Inc., Toronto, ON, Canada). ^e^ Log of the polycratic capacity factor determined as lipophilicity parameter by a reversed phase (RP) HPLC technique, as described in Section 3. ^f^ MM = morpholino-methyl. ^g^ Stable for 4 h in PBS (50 mM, pH 7.4, KCl 0.15 M) and in HCl (0.01 M, pH 2.0, 0.15 M KCl) at 25 ± 1 °C.

**Table 3:** Docking metrics of four diversely R-substituted 1-Ph-DHPIQ derivatives.

N	FEB ^(a)^	ΔE ^(b)^	LE ^(c)^	TAN ^(d)^	POP ^(e)^
**4a**	−8.18	0.65	0.315	0.254	251/1000
**6a**	−7.96	1.04	0.306	0.268	17/1000
**7c**	−11.0	0.08	0.307	0.307	265/1000
**8c**	−10.6	0.21	0.279	0.276	121/1000

^(a)^ FEB, free energy of binding (kcal·mol^−1^); ^(b)^ ∆E, energy difference between the selected pose and the relative global minimum (kcal·mol^−1^); ^(c)^ LE, ligand efficacy; ^(d)^ TAN, Tanimoto–Combo similarity coefficient with elacridar X-ray pose; ^(e)^ POP, cluster members’ population.

## Data Availability

Data are contained only within the article and Supplementary Materials.

## Supplementary Materials

The following supporting information can be downloaded at: https://www.mdpi.com/article/10.3390/ph17040539/s1, Figures S1–S9: 1H and ^13^C-NMR of compounds **4b**, **5a**, **5c**, **6d**, **6e**, **7c**, **8b**, **8c** and **8c**·HCl; Figure S10: Plot of P-gp inhibition potency versus free energy of binding (FEB); Table S1: Squared correlation matrix among biological and physicochemical parameters.
