# Supplementary material for: Nature-Inspired 1-Phenylpyrrolo[2,1-a]isoquinoline Scaffold for Novel Antiproliferative Agents Circumventing P-Glycoprotein-Dependent Multidrug Resistance
Source: Pharmaceuticals (Basel). 2024 Apr 22;17(4):539. doi: 10.3390/ph17040539 (PMC11054433; doi:10.3390/ph17040539)
Supplement: Supplementary file 1 [file pharmaceuticals-17-00539-s001.zip › pharmaceuticals-2928359-supplementary.pdf]

Supporting Information

Nature-inspired 1-phenylpyrrolo[2,1-*a*]isoquinoline scaffold for novel antiproliferative agents circumventing P-glycoprotein-dependent multidrug resistance

Alisa A. Nevskaya [1a], Rosa Purgatorio [2], Tatiana Borisova [1a], Alexey V. Varlamov [1a], Lada V. Anikina [3], Arina Obydennik [1a], Elena Yu. Nevskaya [1b], Mauro Niso [2], Nicola A. Colabufo[2], Antonio Carrieri [2], Marco Catto [2], Modesto de Candia [2], Leonid G. Voskressensky [1a] and Cosimo D. Altomare [2]*

1 [a] Organic Chemistry Department, [b] General and Inorganic Chemistry Department, Peoples' Friendship University of Russia (RUDN University), 6 Miklukho-Maklaya St., 117198 Moscow, Russian Federation
2 Department of Pharmacy-Pharmaceutical Sciences, University of Bari Aldo Moro, Via E. Orabona 4, 70125 Bari, Italy
3 Institute of Physiologically Active Compounds of the FSBIS of the Federal Research Center for Problems of Chemical Physics and Medicinal Chemistry of the RAS, 1 Severnyi Proezd, 142432 Chernogolovka, Russian Federation
* Correspondence: cosimodamiano.altomare@uniba.it

Table of content

^{1}H and ^{13}C-NMR of **4b**	Figure S1	Page 2
^{1}H and ^{13}C-NMR of **5a**	Figure S2	Page 3
^{1}H and ^{13}C-NMR of **5c**	Figure S3	Page 4
^{1}H and ^{13}C-NMR of **6d**	Figure S4	Page 5
^{1}H and ^{13}C-NMR of **6e**	Figure S5	Page 6
^{1}H and ^{13}C-NMR of **7c**	Figure S6	Page 7
^{1}H and ^{13}C-NMR of **8b**	Figure S7	Page 8
^{1}H and ^{13}C-NMR of **8c**	Figure S8	Page 9
^{1}H and ^{13}C-NMR of **8c·HCl**	Figure S9	Page 10
P-gp inhibition potency *vs* FEB	Figure S10	Page 11
Squared correlation matrix among parameters	Table S1	Page 11

Figure S1: ^{1}H and ^{13}C-NMR of (1-(4-methoxyphenyl)-8,9-dimethoxy -5,6- dihydropyrrolo[2,1-*a*]isoquinoline -2- carbaldehyde (**4b**)

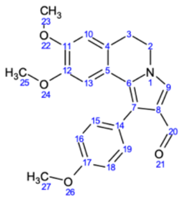

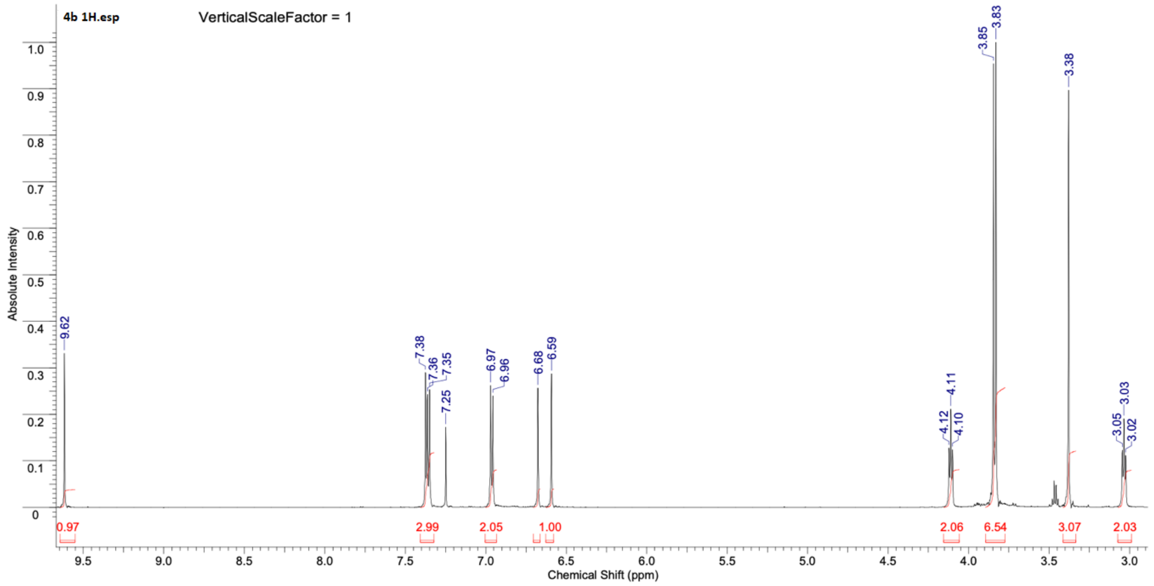

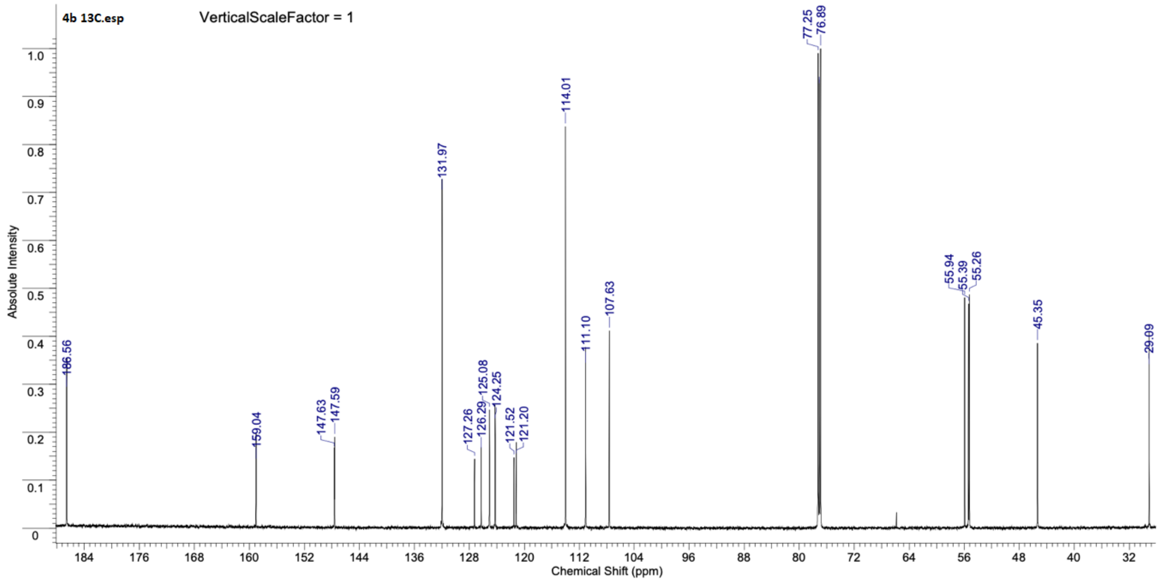

Figure S2: ^{1}H and ^{13}C-NMR of 1-(4-chlorophenyl)-8,9-dimethoxy-5,6-dihydropyrrolo[2,1-*a*]isoquinoline-2-carboxylic acid (**5a**)

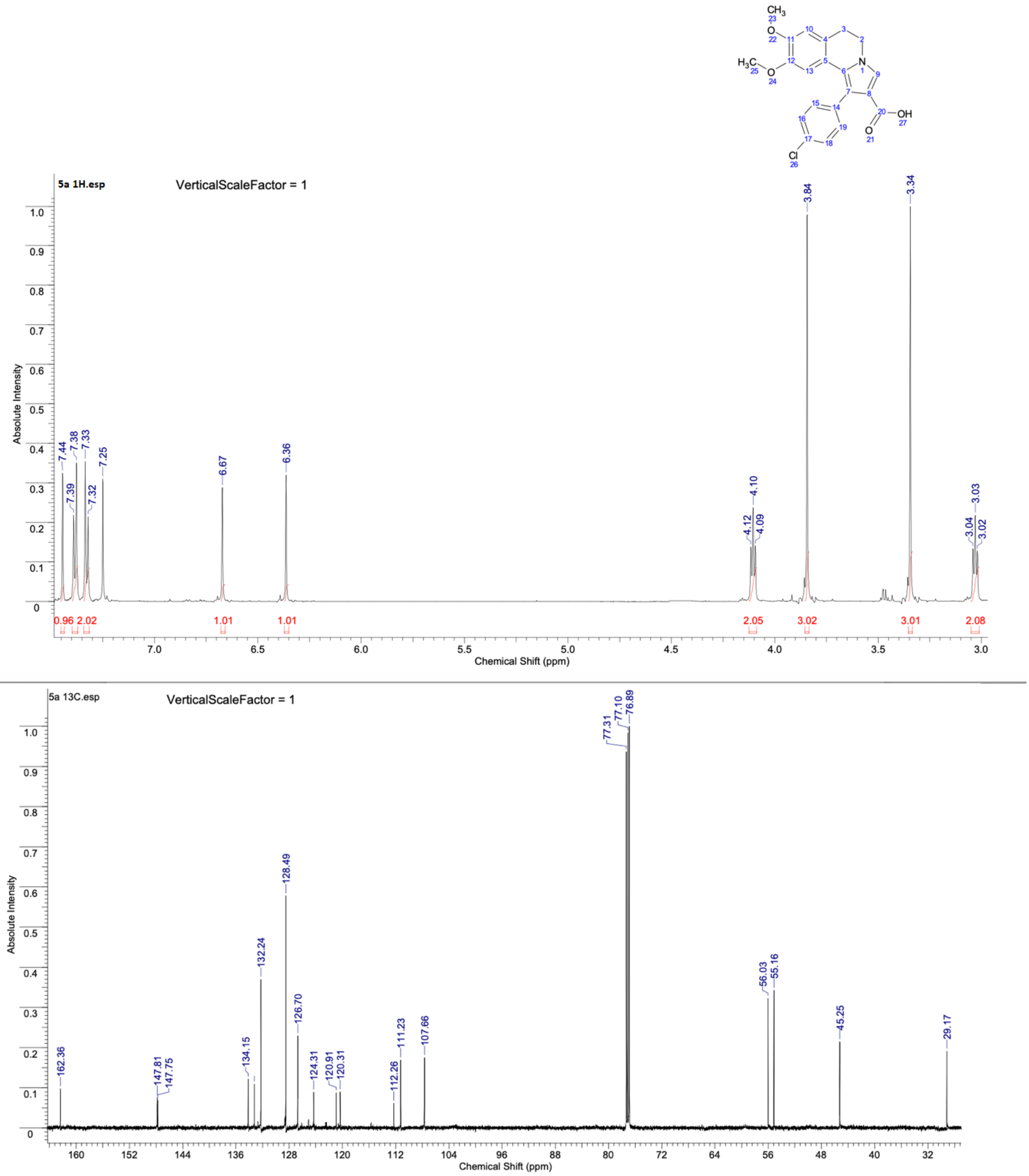

Figure S3: ^{1}H and ^{13}C-NMR of 1-(3,4-diethoxyphenyl)-8,9-diethoxy-5,6-dihydropyrrolo[2,1-*a*]isoquinoline-2-carboxylic acid (**5c**)

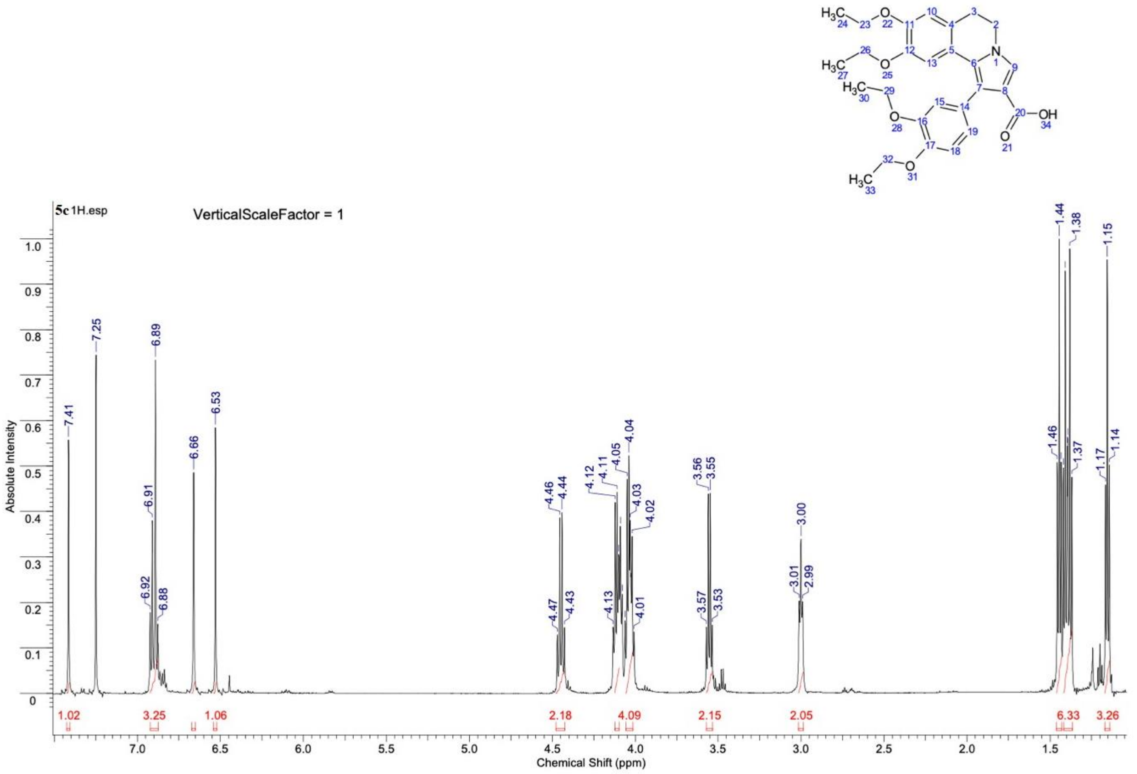

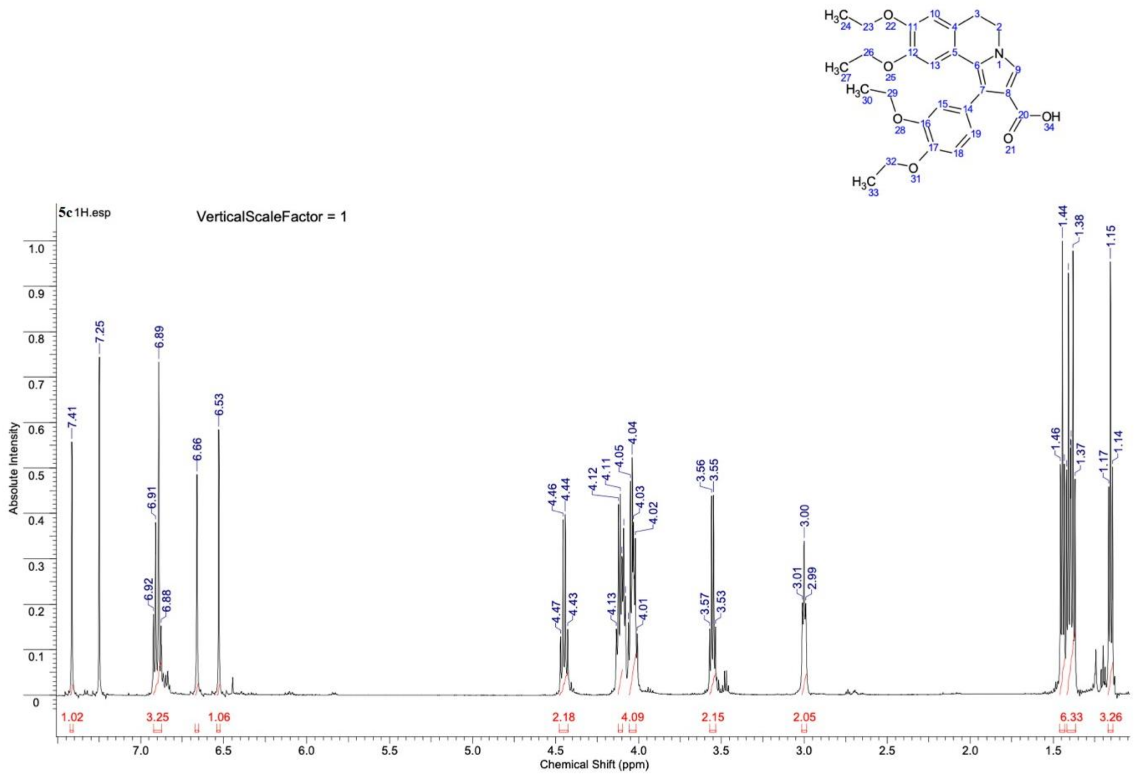

Figure S4: ^{1}H and ^{13}C-NMR of 1-(3,4-diethoxyphenyl)-8,9-diethoxy-3-methyl-5,6-dihydropyrrolo[2,1-*a*]isoquinoline-2-carbonitrile (**6d**)

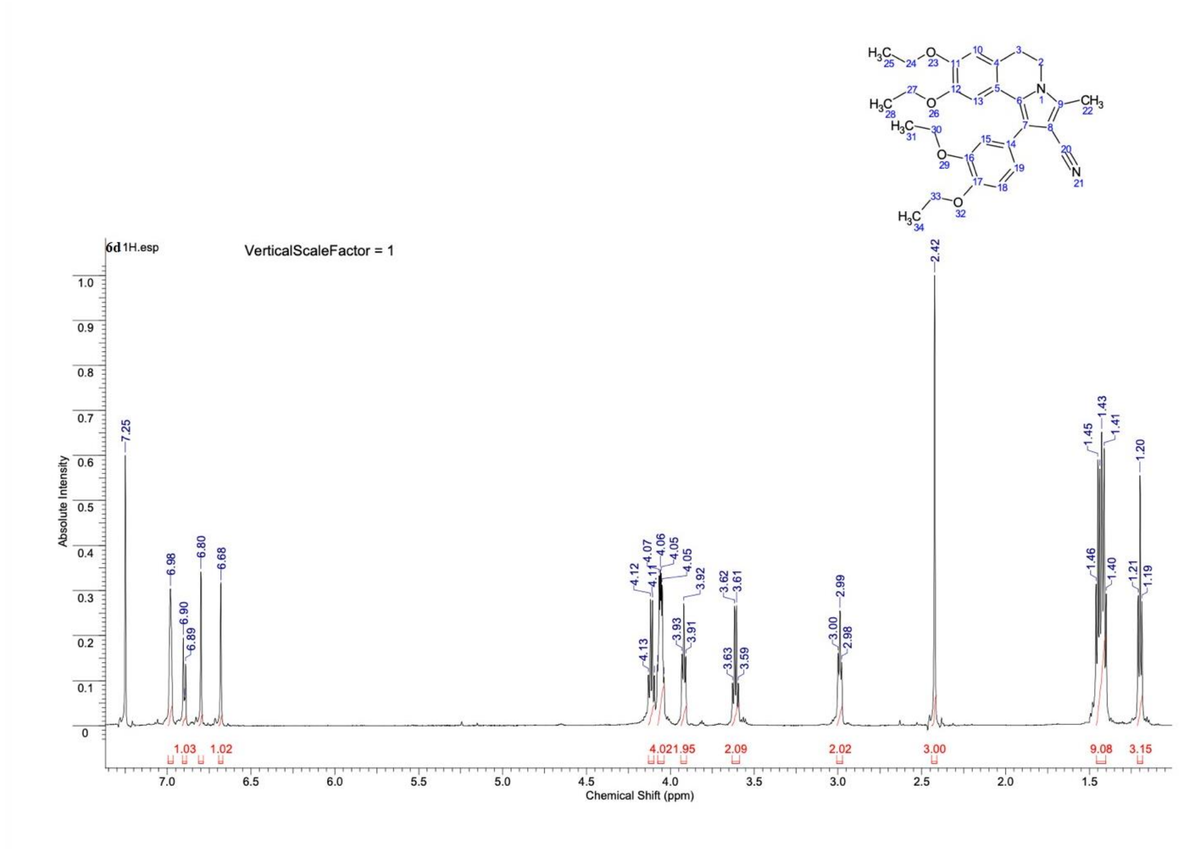

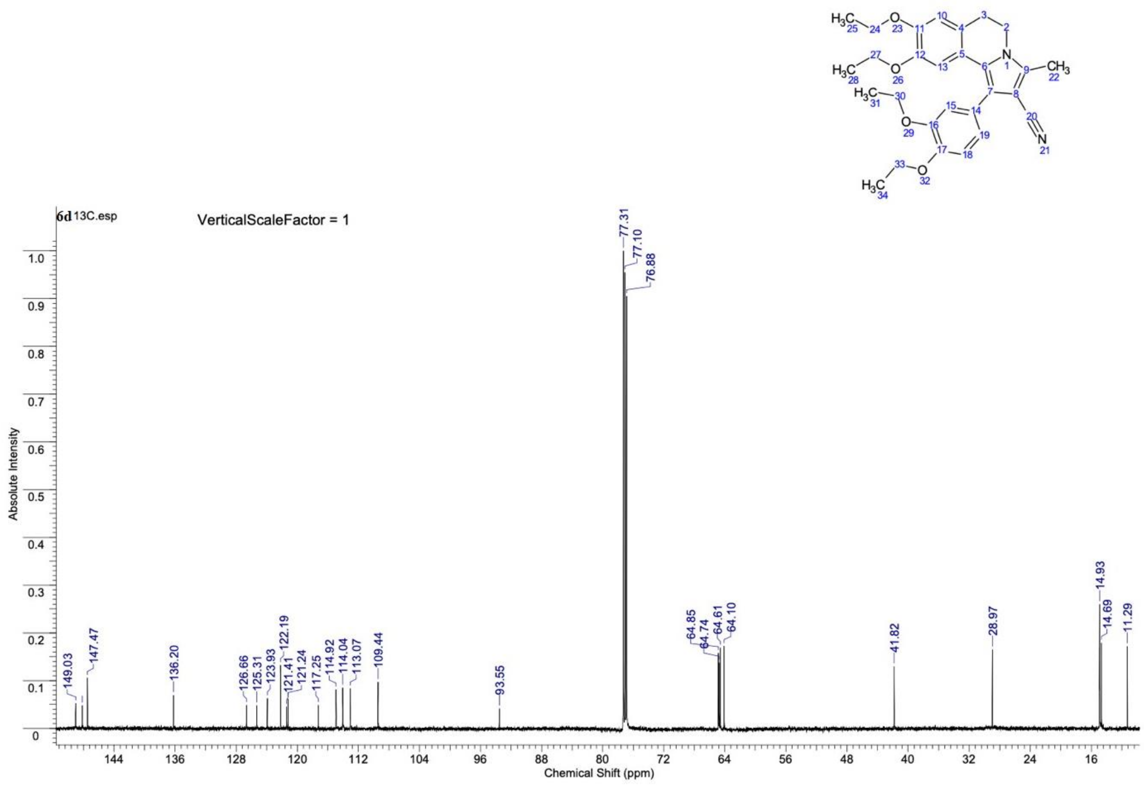

Figure S5: ^{1}H and ^{13}C-NMR of 1-(3,4-diethoxyphenyl)-8,9-diethoxy-3-phenyl-5,6-dihydropyrrolo[2,1-*a*]isoquinoline-2-carbonitrile (**6e**)

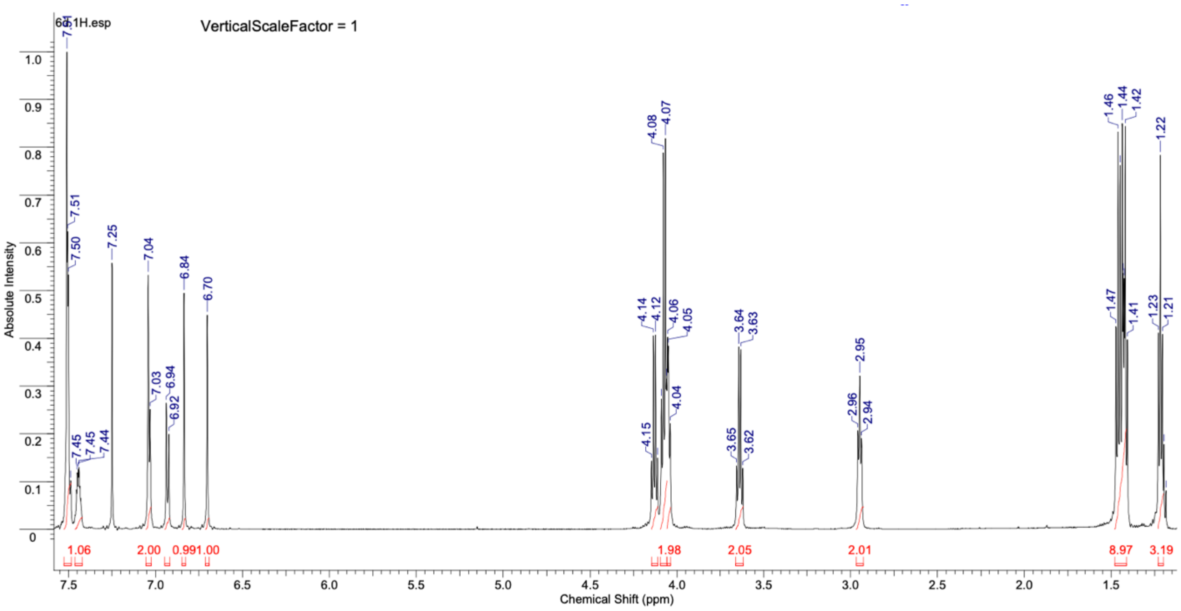

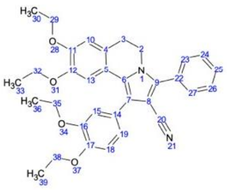

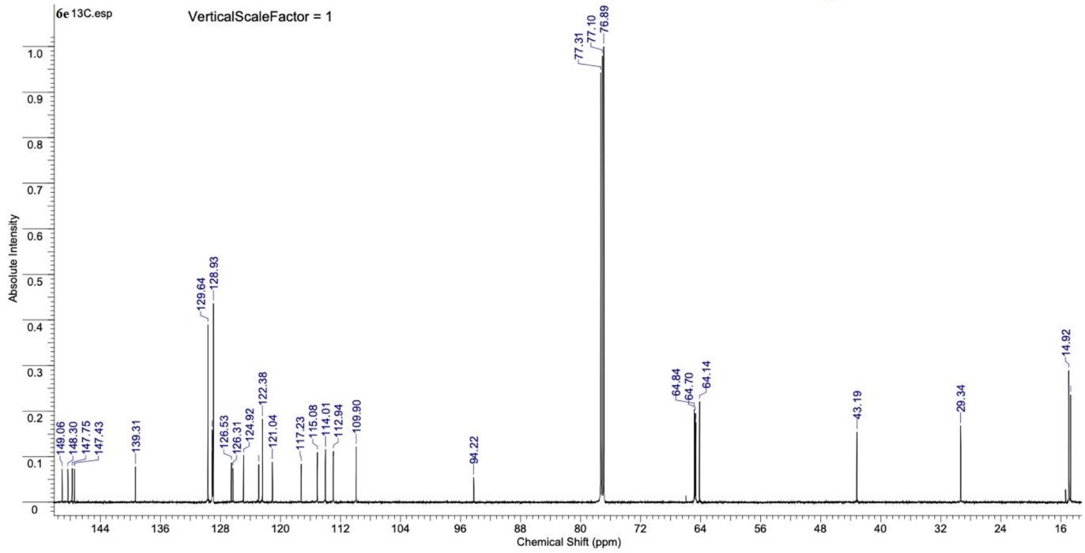

Figure S6: ^{1}H and ^{13}C-NMR of ethyl 1-(3,4-diethoxyphenyl)-8,9-diethoxy-5,6-dihydro pyrrolo[2,1-*a*]isoquinoline-2-carboxylate (**7c**)

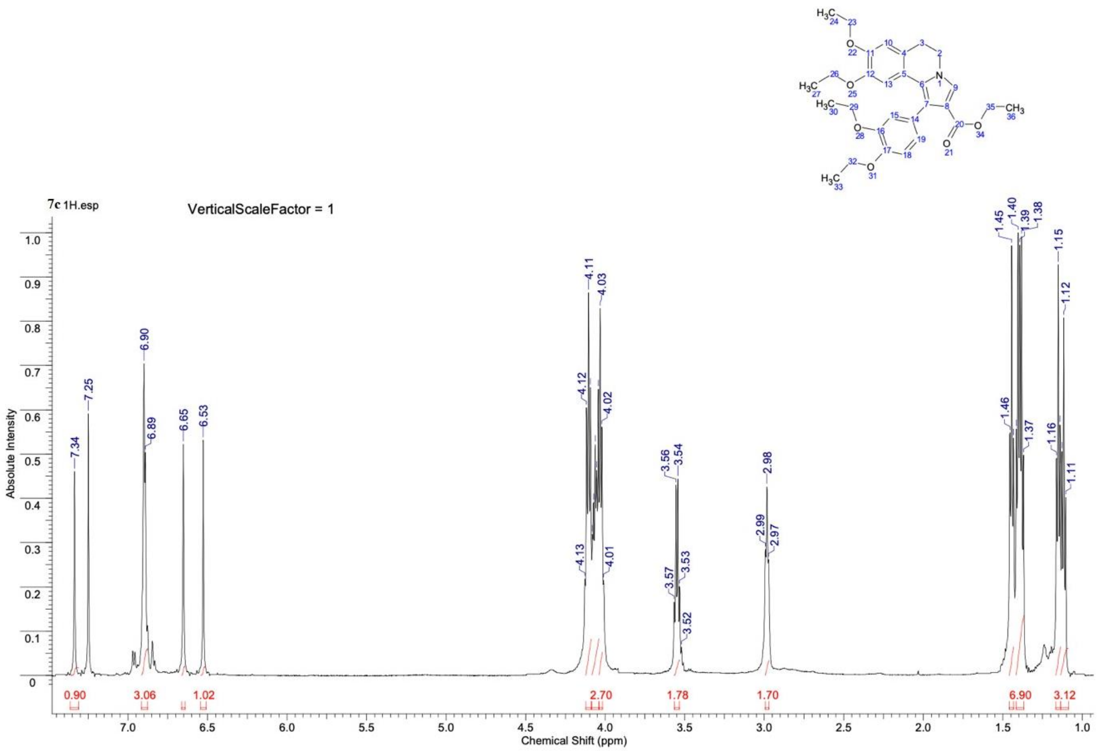

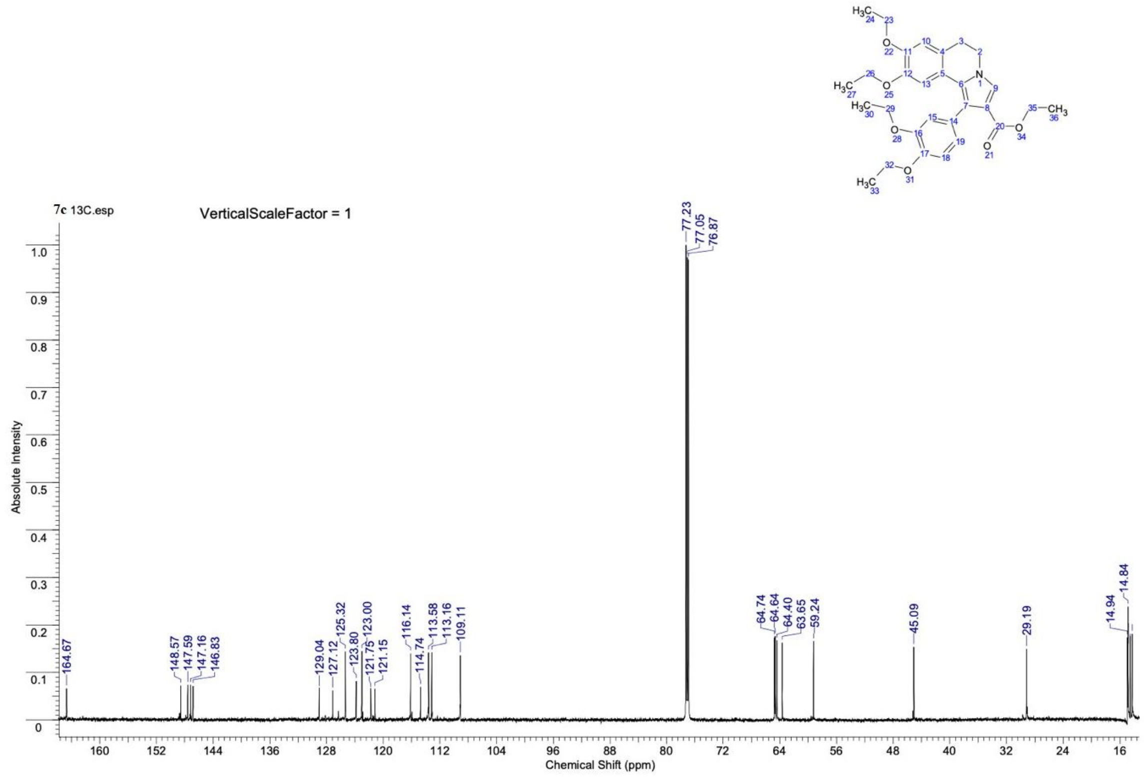

Figure S7: ^{1}H and ^{13}C-NMR of 8,9-dimethoxy-1-(4-methoxyphenyl)-2-(morpholin-4-ylmethyl)-5,6-dihydropyrrolo[2,1-*a*]isoquinoline (**8b**)

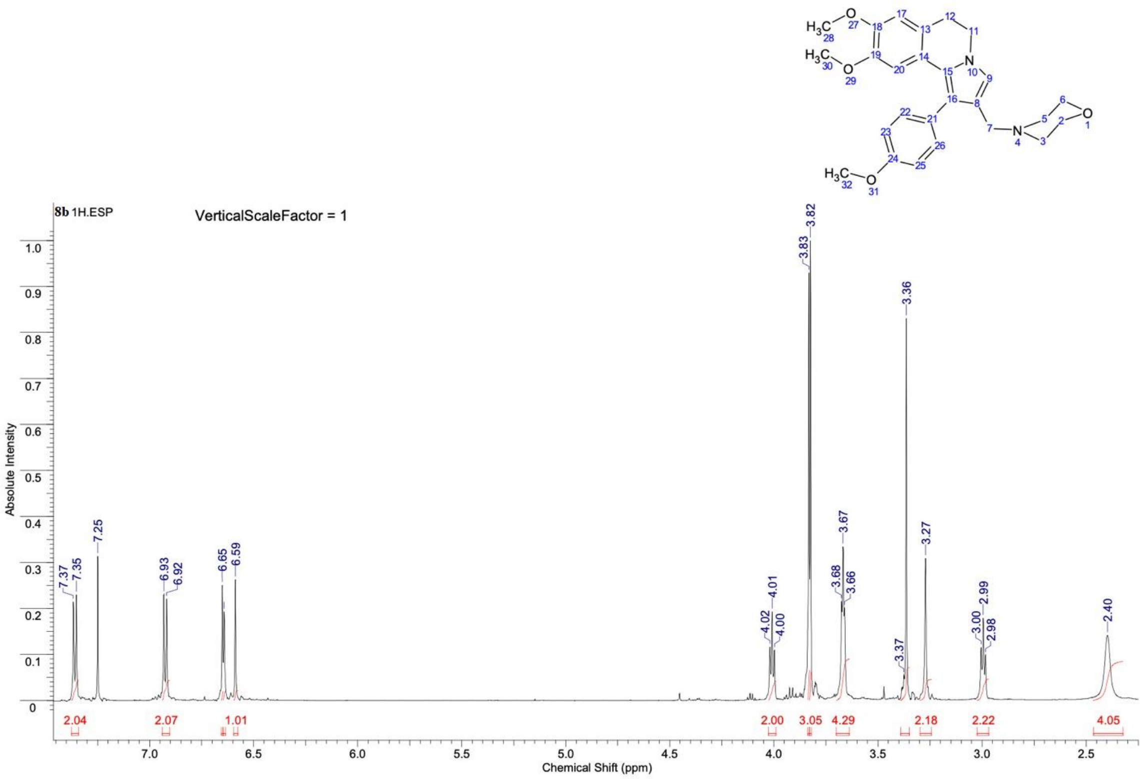

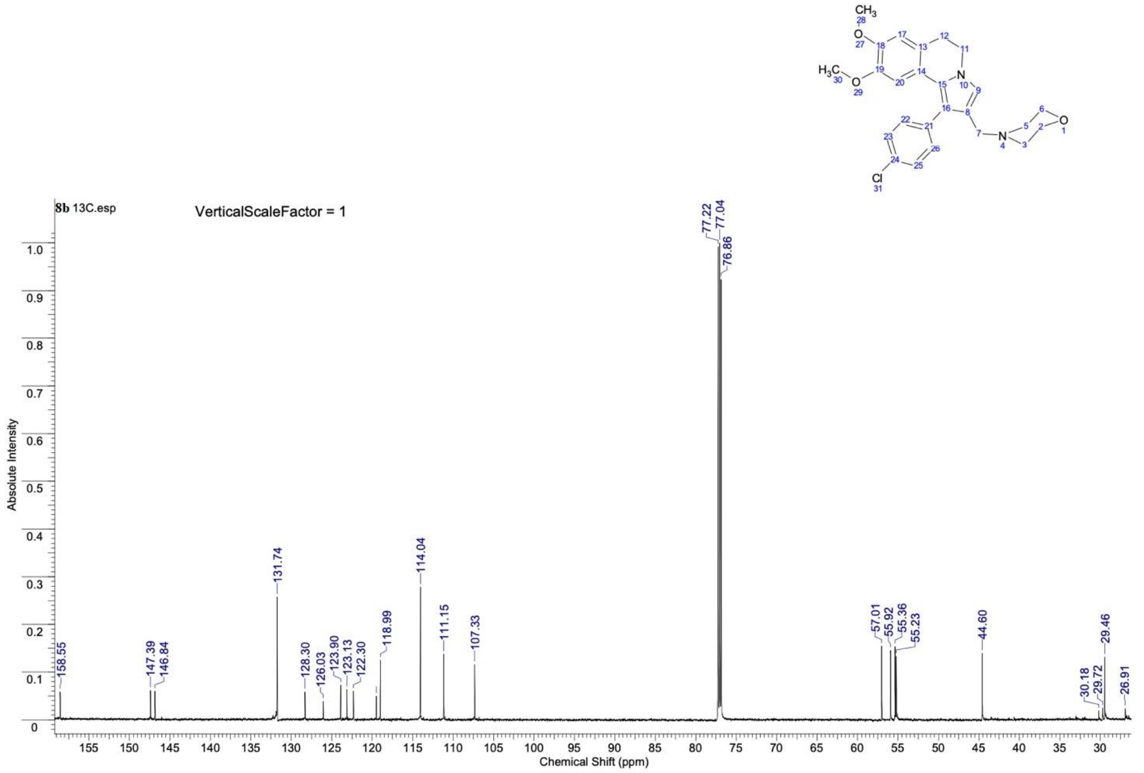

Figure S8: ^{1}H and ^{13}C-NMR of 1-(3,4-diethoxyphenyl)-8,9-diethoxy-2-(morpholin-4-ylmethyl)-5,6-dihydropyrrolo[2,1-*a*]isoquinoline (**8c**)

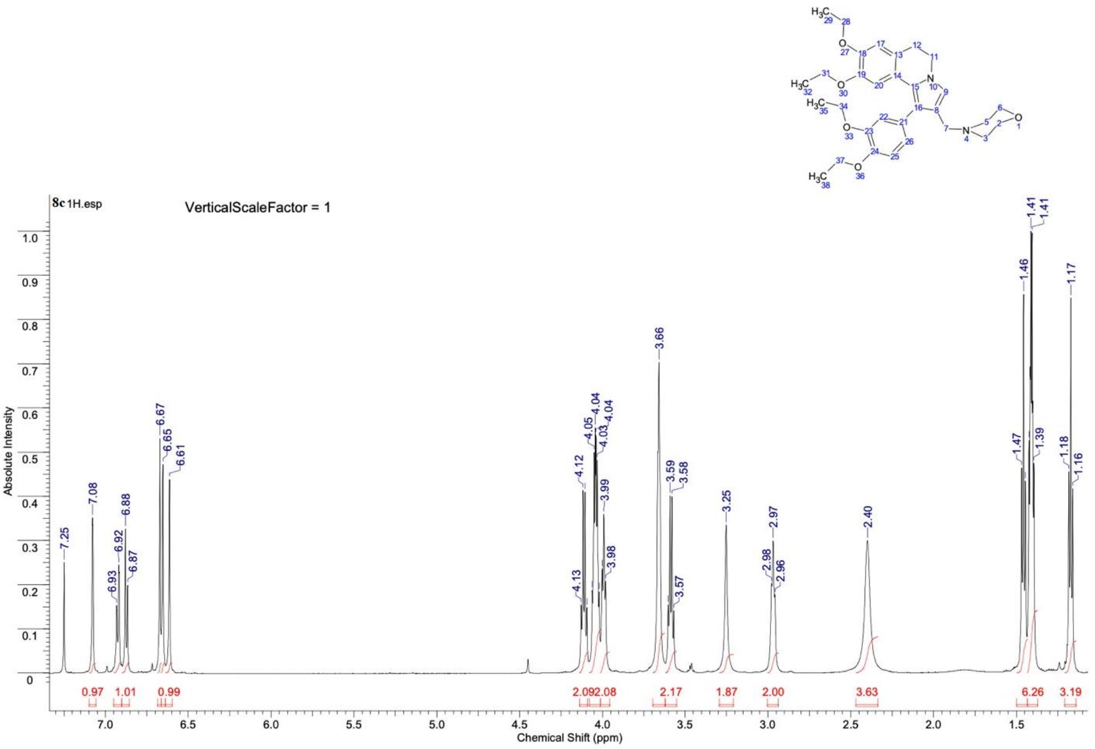

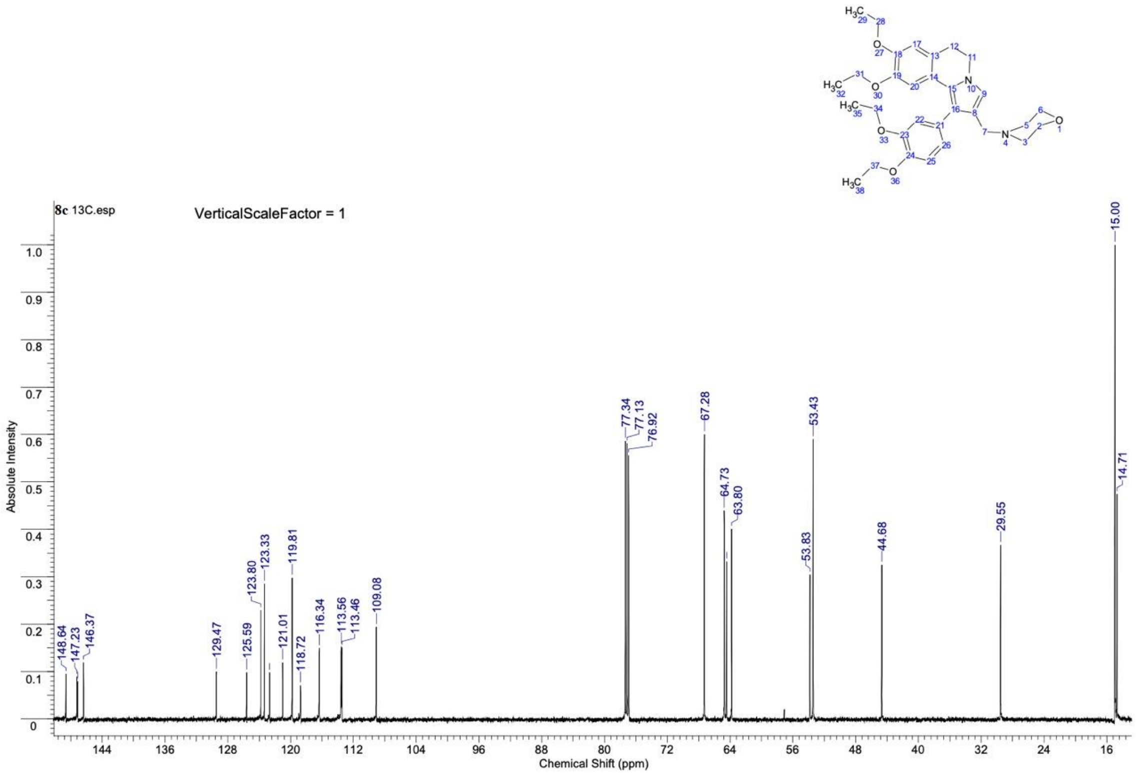

Figure S9: ^{1}H and ^{13}C-NMR of 4-{[1-(3,4-diethoxyphenyl)-8,9-diethoxy-5,6-dihydropyrrolo[2,1-*a*]isoquinolin-2-yl]methyl}morpholin-4-ium chloride (**8c·HCl**)

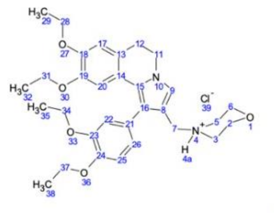

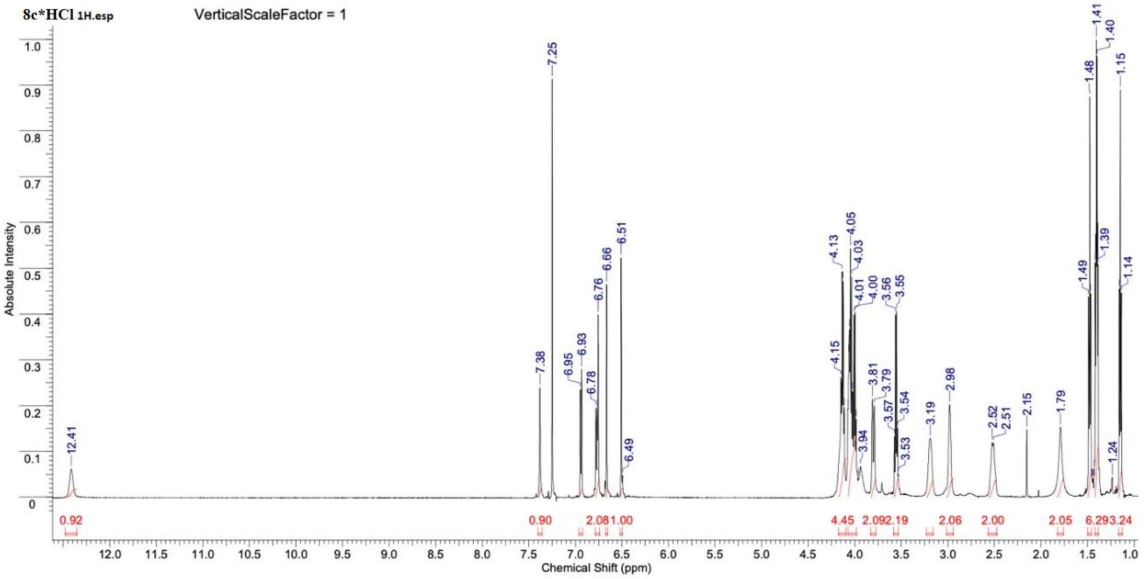

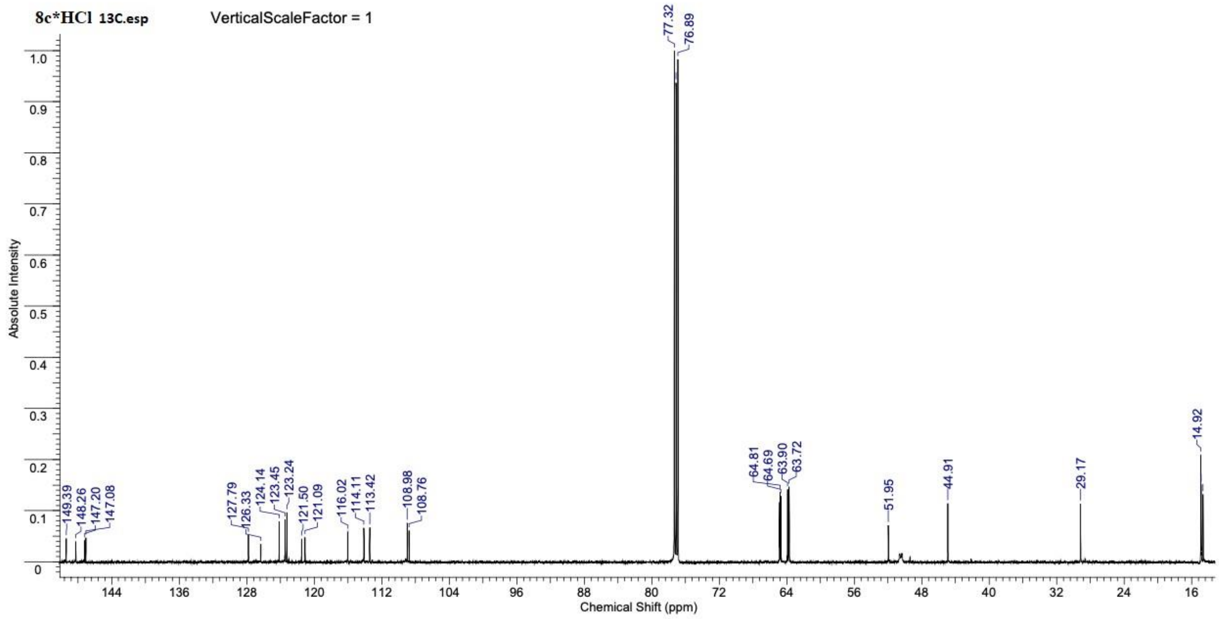

Figure S10. Plot of the P-gp inhibition potency (-log IC_{50}) versus free energy of binding (FEB, kcal·mol^{-1}); data are correlated through a trend of binomial nonlinear relationship (r^2 = 0.879).

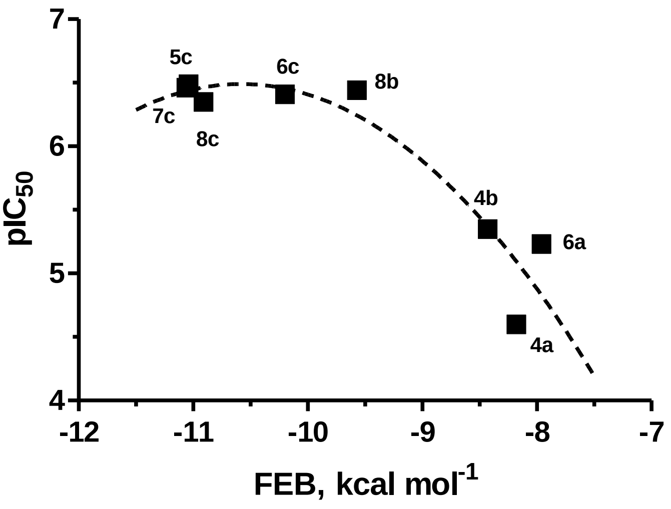

Table S1. Squared correlation matrix (r^2) of the biochemical/biophysical data[a] and physicochemical parameters[b] listed in Table 2.

	P-gp	MRP1	HSA	log S	clog P	log k'_w
**P-gp**	1					
**MRP1**	0.001	1				
**HSA**	0.381	0.284	1			
**log S**	0.147	0.502	0.497	1		
**clog P**	0.255	0.135	0.029	0.017	1	
**log k'_w**	0.237	0.272	0.003	0.041	0.791	1

[a] P-gp (P-glycoprotein) and MRP1 (multidrug-resistance-associated protein-1) inhibition potency data are expressed by -log IC_{50} (mol·L^{-1}) values; for compounds showing IC_{50}s > 100 μM 'truncated' values of -log IC_{50} equal to 4.00 were used in correlation analysis. HSA (human serum albumin) binding affinity is expressed as $\log_{10}$ of the equilibrium dissociation constant (K_D, mol·L^{-1}) determined by a surface plasmon resonance (SPR) technique. [b] Log S is the $\log_{10}$ of the compound solubility (mol·L^{-1}) in PBS (50 mM, pH 7.4, 0.15 M KCl) at 25±1 °C; clog P is the 1-octanol/water partition coefficient calculated by ACDLabs software (release 10.0, Advanced Chemistry Development, Inc., Toronto, Canada); log k'_w is the $\log_{10}$ of the polycratic capacity factor determined by a reversed phase (RP) HPLC technique.
